# A Systematic Review of the Physical, Physiological, Nutritional and Anthropometric Profiles of Soccer Referees

**DOI:** 10.1186/s40798-023-00610-7

**Published:** 2023-08-10

**Authors:** Diogo V. Martinho, Adam Field, André Rebelo, Élvio R. Gouveia, Hugo Sarmento

**Affiliations:** 1 University of Coimbra, Research Unit for Sport and Physical Activity, Faculty of Sport Sciences and Physical Education, Coimbra, Portugal; 2 Laboratory of Robotics and Engineering Systems, Interactive Technologies Institute, Funchal, Portugal; 3 Department of Sport and Exercise Science, Manchester Metropolitan University, Manchester, UK; 4 CIDEFES, Centro de Investigação em Desporto, Educação Física e Exercício e Saúde, Universidade Lusófona, Lisbon, Portugal; 5 COD, Center of Sports Optimization, Sporting Clube de Portugal, Lisbon, Portugal; 6 Department of Physical Education and Sport, University of Madeira, Funchal, Portugal

**Keywords:** Match performance, Physical tests, Nutrition, Body composition, Referees

## Abstract

**Background:**

The importance of soccer referees is widely accepted by international soccer organizations and governing bodies, although there is little research summarizing and appraising the literature on soccer referees. The aim of this study was to systematically review the information related to physical demands, nutrition and physiological profiling of soccer referees.

**Methods:**

Conforming with Preferred Reporting Items for Systematic Reviews and Meta-Analyses (PRISMA) guidelines, searches of three electronic databases (Web of Sciences, PubMed and Scopus) were conducted on 24 April 2022. The following search terms were used: (Soccer OR football AND refer*) AND (physical OR physiolo* OR load* OR “body composition” OR “fat mass” OR “fat free mass” OR “body size” OR “nutrition*” OR “nutritional assessment” OR “nutritional intake” OR “macronutrient*” OR “micronutrient*”). The inclusion criteria of the manuscripts written in English were that articles with male and/or female soccer referees and included relevant data concerning performance, physical testing, nutrition, body composition, body size and/or physiology of soccer referees. The tools developed by the National Institute of Health were used to assess risk of bias according to the study design.

**Results:**

In total, 110 manuscripts were included in the present review. Match activities of soccer referees and assistant referees are not comparable. Variation in performance is influenced by competitive level and competitive schedules. Additionally, match performance is often used to validate field protocols. The associations between match indicators and field protocols were not constant across the included studies, particularly in short-maximal tests. An age decline in physical performance was not conclusive. Body size did not discriminate between referees and assistant referees, as well as referees of different competitive levels. Few studies focused on nutritional guidelines among referees, particularly exercise energy expenditure. Energy intake was comparable across studies, but referees did not follow the daily dietary recommendations. Aerobic output was frequently assessed, and it was not comparable to the values for soccer players.

**Conclusions:**

Although there are decreases in the running profiles of older referees, they maintain the same distances from the ball, and thus, age per se should not be used to define participation at the international level. The assessment of physical fitness warrants future consideration given the levels of fatigue that are apparent at the end of matches. In order to attain ideal levels of body composition, future studies need to provide guidelines for daily energy expenditure and nutritional intake.

*Trial registration*: The protocol was published in INPLASY (International Platform of Registered Systematic Review and Meta-Analysis Protocols) with the registration number 202280052 and https://doi.org/10.37766/inplasy2022.8.005.

**Supplementary Information:**

The online version contains supplementary material available at 10.1186/s40798-023-00610-7.

## Key points

- Age is not a useful indicator of match performance in soccer referees.
- Referees and assistant referees demonstrate distinct activity profiles during matches.
- Field protocols designed for soccer referees are not strongly associated with match performance.
- Nutritional guidelines are generalized from soccer players to referees; however, bespoke studies regarding nutritional guidelines should be developed for soccer referees.

## Background

According to official data from the Federation Internationale de Football (FIFA), in 2006, 843,000 referees and assistant referees were involved in soccer [1]. The Union of European Football Associations (UEFA), English Football League, Premier League and The Football Association created special programs in order to guide training, development and mentoring of soccer referees and assistant referees [2, 3]. Therefore, the importance of soccer referees is widely recognized by international soccer organizations and governing bodies [4, 5]. The FIFA International Refereeing List is a global publication of referees and assistants qualified to officiate at international level [6, 7]. In order to qualify as a FIFA referee, field protocols must be completed [7, 8]. The results of these tests are compared to reference values according to sex and competitive level [7]. The application of these protocols is often generalizable within national institutions [9].

A recent review examined the validity and reliability of fitness tests in European elite soccer referees [10]. This study only included elite soccer referees from Europe and the criteria used to investigate the validity of field protocols were widely variable (i.e. match performance, discrimination by competitive level or age groups and comparisons with concurrent field protocols or physiological outputs). However, other studies that associated match performance indicators [11–14], age [15, 16] and concurrent protocols or physiological outputs [17, 18] with physical tests were not included in the previous review.

An additional question among soccer referees is the age defined by FIFA for forced retirement (i.e. 45 years old). Instead, additional physical tests, medical examinations and technical assessments could be required by FIFA based on an individual analysis of individual referees ≥ 45 years old [7]. However, for example, in Portugal, the national organization [19] defined a narrower age range age for international-level referees (25–37 years) and assistants (31–39 years). Research suggests that older referees tend to cover less total distance and high-speed distance than younger referees, but differences between groups were negligible for distance from the ball [20]. This study suggests that older referees maintained the pacing of the game and perhaps demonstrate tactical superiority to “keep up with play”. A meta-analysis that described match activities in European and South American soccer referees reported that referees covered, on average, 10 km per game with approximately 37% of the distance covered was spent running at lower intensities [21]. Considerable levels of heterogeneity were noted across the studies thereby, the results should not be globally extrapolated for all soccer referees [21]. Nevertheless, referee activities during the matches are associated with team strategies (i.e. tactical positioning on the field) [22, 23] and this issue was neglected in the analyses of the mentioned review [21].

Interestingly, size, body composition and nutritional guidelines which are related to match indicators and physical conditioning are often overlooked as criteria for soccer official selection. Height was negatively associated with disciplinary behaviour, with shorter soccer referees producing more yellow cards [24]. From 2001 to 2012, a significant reduction in body fat percentage was observed in elite Spanish soccer referees [25]. A recent study presented the nutritional data of FIFA soccer referees selected 2012–2013 World Cup [26], with such findings requiring further attention. The guidelines developed for soccer players might be applied to soccer referees should there be a lack of understanding of nutritional requirements among soccer referees. Therefore, further understanding of the current nutritional intakes and habits as well as the energy requirements of soccer referees is needed before bespoke nutritional guidelines can be developed.

Given the discrepancies across studies included in the review that examined the external loads of soccer referees [21], and considering the importance of field protocols in the selection process of international referees, a clear appraisal of the current soccer referee literature is needed for the scientific and applied communities to enhance the scope and application of future research. The aim of this study was to systematically review the information related to the physical demands, nutrition and physiological profiling of soccer referees.

## Methods

The current review was conducted according to Preferred Reporting Items for Systematic Review (PRISMA 2020 statement) following Cochrane Guidelines [27, 28].

### Eligibility Criteria

Empirical research published in peer-review journals was analysed. The publications included in the present review met the following inclusion criteria: (1) male and/or female soccer referees and/or assistant referees; (2) all types of interventions or exposures; and (3) relevant data concerning body size, body composition, physical performance, physiological profiles and nutrition. Given the interest in examining size and composition, manuscripts that described sample characteristics, even though covering different topics (e.g. injuries), were also considered in the present study. Only English publications were assessed.

### Information Source and Search Strategy

Three electronic databases (Web of Sciences, PubMed and Scopus) were searched on 24 April 2022 using the following search terms: (Soccer OR football AND refer*) AND (physical OR physiolo* OR load* OR “body composition” OR “fat mass” OR “fat free mass” OR “body size” OR “nutrition*” OR “nutritional assessment” OR “nutritional intake” OR “macronutrient*” OR “micronutrient*”).

### Selection Process

Reference managing software (EndNoteTMX9, Clarivate Analytics, Philadelphia, PA, USA) was used to export the papers. Duplicates were manually removed and two independent authors (DVM/HS) screened according to the title and abstract. Full-text manuscripts were assessed to check if they met eligibility criteria. A third independent reviewer (AR) was consulted to solve the disputes between authors.

### Data Collection Process and Data Items

Eligible studies were individually examined and extracted by two authors (DVM/HS). Relevant information, including, sampling characteristics, country, purpose of the study, significant results and practical applications, was organized on an adapted template of Cochrane Consumers and Communication Review Group [29]. The studies were, subsequently, categorized into four different topics: size and body composition, performance, physiological variables and nutrition. Few studies included female referees; hence, results were not separately presented by sex. Corresponding authors were contacted where relevant data were not presented within the manuscript.

### Risk of Bias

Three different tools, according to study design, were used to analyse the risk of bias of manuscripts in the present review. The Quality Assessment Tool for Observational Cohort and Cross-Sectional Studies [30] includes fourteen items. These criteria related to the research question, study population, groups recruited from the same population and uniform eligibility criteria, sample size justification, exposure assessed prior to outcome of measurement, sufficient timeframe to observe an effect, different levels of the exposure effect, exposures measurement, repeated exposure assessment, outcomes measurement, blinding of outcomes assessors, follow-up rate and statistical analysis. A global assessment of the publication was undertaken and each article was categorized as good, fair or poor. The Quality Assessment Tool for before and after (pre–post) studies with no control group [31] contained twelve questions about study purpose, inclusion criteria and population, eligibility of participants, sample size, description of intervention, data quality about dependent variables, blinding process, follow-up rates, data analysis, multiple outcome measures, inter and intra-individual variability in addition to a global assessment (good, fair or poor). Finally, the bias of studies with a control group was obtained using a specific tool—Quality Assessment of Controlled Intervention Studies [32]. The tool considers fourteen questions about randomization design, allocation of participants, blinding process, sample characteristics at baseline, dropout, adherence, confounding interventions, measurements of the outcome, power calculation, pre-determined outcomes and intention-to-treat effects. The overall assessment of study quality (good, fair, poor) was assessed via discussions among researches. Two independent authors (DVM/HS) familiarized with the tools completed the bias assessment for each study and disagreements were solved by a third reviewer (AR).

## Results

### Study Selection

**Fig. 1:**
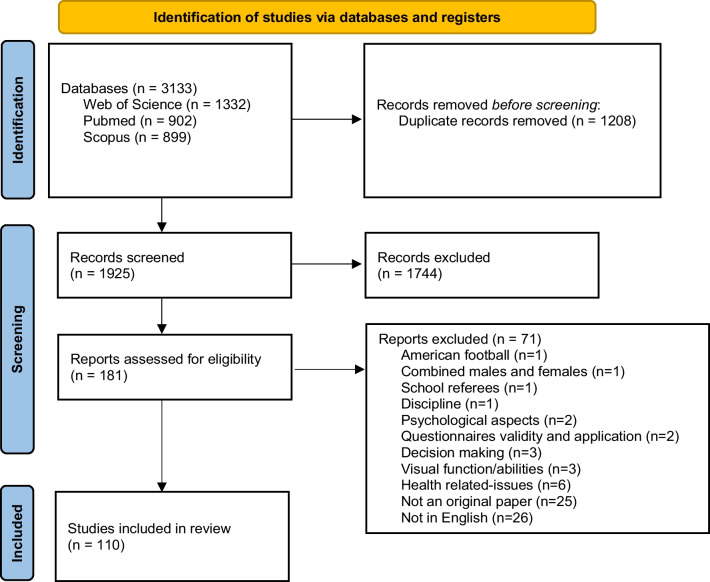
Flow chart of the study selection procedures used in the present review article

The initial search of Web of Science, PubMed and Scopus identified 3133 manuscripts. Duplicates (*n *= 1208) were removed, and subsequently, 1925 titles and abstracts were screened, resulting in the removal of 1744 papers. The full texts of 181 manuscripts were examined in more detail; 71 of which were omitted as eligibility criteria were not satisfied. Many reasons were identified to exclude studies in the present review, including studies with American Football referees (*n *= 1) or school soccer referees (*n *= 1), studies combining males and females (*n *= 1), studies lacking relevance to discipline of soccer referees (*n *= 1), studies assessing psychological aspects (*n *= 2), studies using questionnaires to collect data (*n *= 2), studies about decision making (*n *= 3), studies that evaluated visual function abilities (*n* = 3) and/or studies that investigated health-related issues (*n *= 6). An additional 25 records were excluded due to not being original papers and 26 were not written in English (Fig. 1). Finally, the 110 full texts that met the inclusion criteria were read.

### Study Characteristics

**Fig. 2:**
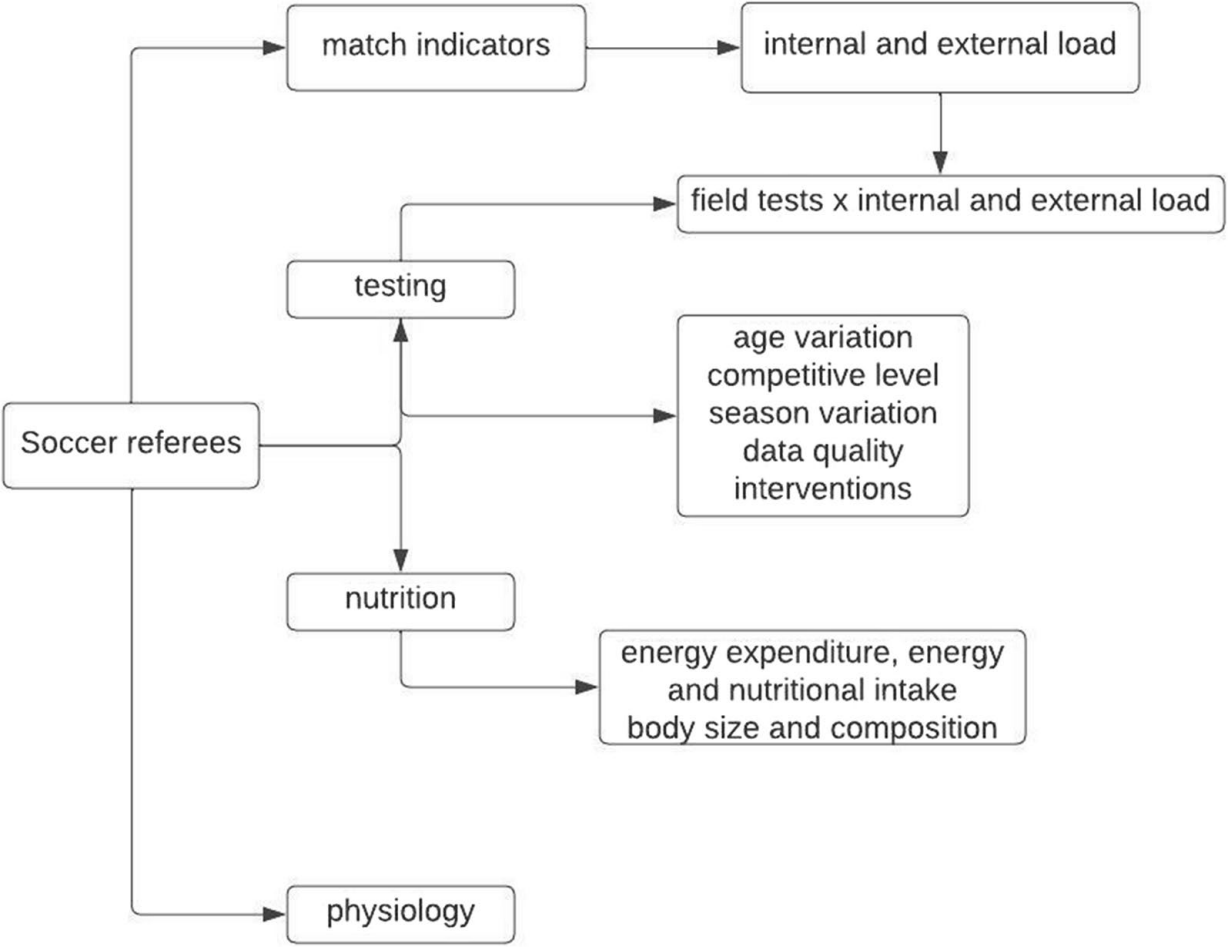
Scope of the literature on soccer referees

The sample characteristics and purpose of each study are summarized in Additional file 1: Table S1. It was decided that the presentation of results should be categorized according to the main purpose of each study. Therefore, as shown in Fig. 2, four topics were considered: match indicators, physical testing, nutrition and physiology. Internal and external load was frequently associated with field-based protocols [12, 13, 22, 33–41]; therefore, studies that examine the relationship between match performance and physical tests are discussed in the 'Physical Testing' section.

Some studies involved exploring the effects of age variation in physical assessments [15, 16, 42–44] and examined variation by competitive levels in soccer referees [43, 45, 46]. Three studies analysed differences in physical performance among adult referees grouped into different age groups [15, 16, 42]. Variations in linear straight sprinting tests, agility tests and YOYO protocol were compared between referees aged ≤ 35 or > 35 years old [43]. The effects of age on field tests were also assessed by examining the correlation coefficient between variables [44]. Comparisons by competitive level were mainly detailed in Italian and Spanish referees [45, 46]. Relationships between different field protocols or data quality concerning physical tests were often described among referees [8, 17, 46–48]. Other studies highlighted seasonal variation in physical tests focused on YOYO protocol and velocity [16, 49]. Interventional studies involving tests were applied on match day [50–53] and training sessions [13, 54–57]. The effects of match load in soccer referees were evaluated on linear velocity [51], jumping performance [52] and isokinetic strength [53]. Three studies confirmed the importance of planning and implementing high-intensity interval training protocols in soccer referees [54, 55, 57]. One randomized cross-over trial examined the re-warm up effects on the YOYO protocol after the application of an intermittent test—The Loughborough Intermittent Shuttle test [58].

Nutrition studies were related to about body size [24, 59, 60], body composition [60–67], energy expenditure [68–71], energy, macronutrients and supplementation intake [72–76]. Daily energy or exercise expenditure was estimated by mathematical equations in two studies  [68, 70] or using training watches [71]. Different methods of body composition were also used to obtain fat mass percentage (i.e. skinfolds, bioimpedance and dual-energy X-ray absorptiometry) [40, 61–64, 66, 67, 77].

Studies investigating the physiology in soccer referees described heart characteristics [78–80], maximal oxygen uptake [17, 81–85] and compared strength in referees across different professional levels [86, 87].

Eight studies reported body size or body composition [88–95]; thereby, the full text was read, and sample characteristics were extracted for the present review. The main results and aims of the aforementioned studies are summarized in Additional file 2: Table S2.

### Risk of Bias

The risk of bias was examined using specific tools according to study design—Quality Assessment Tool for Observational Cohort and Cross-Sectional Studies [30], the Quality Assessment Tool for Before–After (Pre–Post) Studies With No Control Group [31] and Quality Assessment of Controlled Intervention Studies [32] as shown in Additional file 3: Table S3. In general, cross-sectional, cohort and pre–post-studies were classified as good or fair even though sample size estimation was lacking. Few observational studies included more than one observation. None of the pre–post-studies considered intra-individual variability to determine the effects at the group level. Two interventions with a control group were considered in the present analysis [58, 96]. The latter did not clarify if the sample was randomized and how the allocation of participants was conducted.

### Results of Individual Studies

#### Match Indicators

The match indicators among soccer referees are described in Table 1. Although intermittent activity during a soccer match was noted in referees [34] and assistant referees [36], they varied in distances covered and activity profiles [13, 89, 97]. Contrasting match activities of referees and assistant referees in the American Cup showed that referees covered 4.4 km more and greater distance in accelerations than assistant referees [97]. The total distance and distance in high-intensity running covered by referees were 10.3 km and 1.9 km, respectively. Corresponding data showed that assistant referees covered, on average, 6.8 km during a match and 1.0 km at high intensities [89]. Match performance in the studies was determined by a considerable number of factors: total distance covered during a game [98], activities of the players [5, 99], distance covered by the ball [22], variability between matches [100], competitive level [5, 101–104], age [20] and competitive schedule [105]. Distance from the ball and infringements was used to examine the capacity of referees to maintain the pace of the game [20, 34, 89, 100, 106]. The distance from the offside line was frequently reported to investigate the performance of assistant referees [23, 36, 107]. Assistant referees maintained the distance from the offside line across the entire match [36, 107]. Only two studies examined female referees [108] and assistant referees [107]. Female referees covered, on average, 10 km per match and spent 7% and 21% of the time walking and jogging, respectively [108]. In contrast, assistant referees covered 5.6 km per game and sideways movements were the most representative activity during the match [107].

**Table 1:** Studies focused on match indicators

Match indicators		
Study	Results/main findings	Practical applications
Catterall et al. [109]	Referees covered 8–10 km, significantly less distance during the second half compared to the first half. The mean heart rate was 165 beats per minute, corresponding to 95% of the maximal heart rate. Jogging, walking and running backwards were the most common activities during the game. 1.1 km was covered sprinting	Training programs should include aerobic and anaerobic activities
Johnston and McNaughton [110]	Soccer referees covered, on average, 9.4 km per game, which included walking (18.9%), jogging (46.6%), running/striding (12.1%), sprinting (6.2%) and backward movements (16.2%). Most of the time was spent at low intensity (65.5%), but the heart rate was above 85% of the maximal heart rate without differences from the first to the second half	Training programs should include aerobic and anaerobic activities
D’Ottavio and Castagna [111]	Referees spent 17.2% of the total match in high-intensity activities (sum of actions faster than 18 km^−1^). Differences between the first and second half were considerable. For example, referees covered less distance during the second half on medium-intensity runs, walking forward and walking backwards and spent more time standing. However, the high-intensity distance was comparable, and the total distance covered during the first and second half was 5854 m and 5612 m, respectively	Training programs should combine aerobic and high-intensity activities in training programs—maximal intensities should not exceed 30 m
D’Ottavio and Castagna [11]	The mean heart rate was 163 beats per minute (89.1% of the maximal heart), and no changes were noted between the first and second half. Approximately 42% of the time was covered at velocities higher than 13 km h^−1^	The internal and external load analysis showed the intermittent pattern of soccer activities in referees. The time of sprints ranged between 2 and 4 s
Krustrup et al. [34]	The distance covered during a match was approximately 10 km: 3.87 km walking, 1.94 km jogging, 1.69 km low-speed running and 1.67 km high-intensity running. The last parameter decreased from the first to the second half. Distance from infringements was, on average, 11 m, increasing from the first to the second half. Mean heart rate and blood lactate were 162 beats per minute (85% of maximal heart rate) and 4.9 mmol l^−1^, respectively. The total high-intensity activities were related to the distance covered in YOYO protocol. The intermittent exercise intervention had an impact on the high-intensity distance covered (after: 2.06 ± 0.13 km; before 1.69 ± 0.08 km) and heart rate (after: 159 ± 1 beat per minute; before:164 ± 2 beats per minute)	see match indicators × testing and Table 2—interventions section
Krustrup et al. [13]	The distance covered by the assistant referees was, on average, 7.28 km. Low and high-intensity running activities corresponded to 19.3% and 4.1% of the total time. Distances covered walking, jogging, low-speed running, backwards running and sideways running were 3.1 km, 1.0 km, 0.8 km, 0.06 km and 1.16 km, respectively. The mean heart rate during the match was 173 beats per minute (73% of maximal heart rate). Blood lactate was 4.7 and 4.8 mmol l^−1^ after the first and second halves. Maximal oxygen uptake was, on average, 3.71 l min^−1^	Aerobic and anaerobic activities are part of assistant referee activities. Sideways and forward running combined with intermittent exercise training need to be included in training programs
Castagna and Abt [98]	Considering different distances covered during matches (short vs. long), no differences were reported in high-intensity activities. Increments in submaximal activities determined the distance covered on long matches. This issue also highlights the importance of high-intensity activities in short and long matches	Soccer referees should include aerobic activities as well high-intensity intermittent exercises
Castagna et al. [101]	International referees (approximately 11 km) covered less distance than national referees (approximately 13 km) and also performed less distance at velocities faster than 18 km^−1^	Given the relationship between distance covered and positioning during the match, training programmes for international soccer referees need to be adjusted
Helsen and Bultynck [112]	The mean heart rate for referees and assistant referees was 155 and 140 beats per minute, respectively, corresponding to 85% and 77% of the maximal heart rate. Among referees, during the match, the percentage of maximal heart rate tends to increase in the first and second half. In contrast, in assistant referees, heart rate tended to decrease in the first half, which is maintained relatively stable during the second half. Most of the total time was spent in maximum efforts and high-intensity activities, while assistant referees spent most of the total time in low-intensity activities. Referees made 137 observations per game	High-intensity intermittent protocols should be part of training programs—aerobic components should be prioritized. Additionally, video analysis should also be included in training programs
Weston et al. [113]	A significant relationship between heart rate and rate of perceived effort was significant. In parallel, heart rate and perceived effort were significantly higher in Premier League soccer referees compared to Football League. Within each league, referee experience did not affect heart rate and rate of perceived effort	The results of this study showed that heart rate and rate of perceived effort were valid measures of match intensity
Weston et al. [5]	The values of high-intensity running and total distances reported in the first half were related to the values of the second half. No significant changes in these parameters were noted across the season. Match-to-match variation revealed that referees covered less distance at high-intensity running during the second half	Match activities of soccer referees are related to player demands. An appropriate schedule should be created in order to permit an appropriate recovery from match to match
Mallo et al. [36]	Assistant referees covered, on average, 6.1 km per game. Comparisons between the first half and second half revealed that distance tends to decrease from the first (3.2 km) to the second half (3.0 km). Regarding match-specific activities, assistant referees were 56.9% of total playing time still and 24.1% walking. In addition, 1.4% of playing time was spent sprinting. During the second half, assistant referees passed more time still and decreased the time spent jogging and running. Sideway movements were frequent in assistant referees (30% of the total distance). Distance from offside situations (*n *= 81) was, on average, 1.36 m	Specific training sessions for assistant referees should be developed
Krustrup et al. [89]	Referees and assistant referees differed in total distance covered (referees: 10.3 km; assistant referees: 6.8 km) and high-intensity running (referees: 1.9 km; assistant referees: 1.0 km). A considerable distance of backward running was noted for referees (0.9 km), while assistant referees tended to perform sideways running (1.5 km). The mean heart rate was higher for referees than assistant referees; no difference was found in blood lactate. High-intensity running did not decrease across the games, and this parameter was related to the distance from infringements	High-intensity running was frequent among international referees. The current study noted substantial differences in match performance between referees and assistant referees, and this point needs consideration in developing training programs
Mallo et al. [22]	The mean distance covered during the games was 10.2 km. The total ball distance covered was partially related to high-intensity activities and the total distance covered by the referees. In the current study, the mean heart rate was 161 beats per minute, corresponding to 86% of the maximal heart rate. Mean heart rate is not helpful—comparisons between high-intensity efforts and heart rate should be adjusted for narrow intervals (i.e. 5 min). Based on 304 incidents, the mean distance was 16.3 m	The activity is related to the distance covered by the ball and, consequently, by the playing style of each team. Heart rate analysed in narrow intervals is a good tool for determining high-match intensity
Mallo et al. [23]	Assistant referees covered, on average, 5.8 km and spent 29.7% of playing time in sideways movements. Compared to data from the under-17 World Championship 2003, assistant referees of the Confederations Cup covered more distance at higher speeds. Ball distance was related to the distance covered by the referees and the frequency of high-intensity activities. Distance from the offside line was, on average, 0.64 m, and the mean heart rate was 140 beats per minute	Team playing style, as well as competitive level, influence the activity of assistant referees
Catteeuw et al. [114]	From 4960 offside situations, 868 (17.5%) situations were marked as errors (737 non-flag errors and 95 flag errors). Considering six 15-min match periods, the error distribution is constant across the game. No significant associations were noted between the errors and the assistant referee position in relation to the offside line and movement speed. Note that most of the offside decisions were taken walking or jogging. The optical error hypothesis (position of attacker and referee about the second defender) was not confirmed, and non-flag errors were observed in flash-lag situations	Decision-making training can be helpful using defenders and attackers on the field. Another essential tool is video feedback, and off-field offside decision-making tests and programs need particular attention
Mallo et al. [107]	The distance covered by the assistant referees was 5.6 km, and sideways movements were the most representative activity during the soccer match (26.6%). High-intensity activities (velocity > 13 km^−1^) accounted for 2.0 km, and the frequency of actions was stable across the matches. The distance from the offside line was, on average, 1.06 m	Male and female assistant referees are comparable in terms of match activity patterns. Since the distance from the offside line is similar across the game, the results of this study indicated that female assistant referees involved in the under-20 Women World Championship could maintain the pace during the match
Mallo et al. [108]	Female referees covered, on average, 10 km per match. The mean distance decreased from the first half (5.2 km) to the second half (4.9 km). 25.7% of the time is spent walking, while medium-intensity activities (i.e. jogging) accounted for 21% of match time. High-speed activities accounted for 6% of the total time. No differences were noted from the first to the second half. No variation was noted in the distance from incidents and ball position from the first to the second half	Female referees physical demands are closely related to male referees (moderate competitive level). The female participants that participated in the under-20 Women World Championship maintained the pace of the game
Weston et al. [20]	Total distance covered during the match, high-intensity running and sprint frequency negatively correlated with age. On the other hand, the distance from the ball and fouls were not related to age	The reduced physical match activities associated with increasing age did not impact the positioning of referees. Consequently, the age of retirement (45 years old) should be reviewed
Di Salvo et al. [115]	Referees and assistant referees covered, on average, 11.6 km and 6.5 km. Jogging and high-speed running were performed less during the second half. The walking activity was frequent in the second half compared to the first half	Although variation across competitions has been noted, the present study confirms the importance of short-maximal intensities among soccer referees
Weston et al. [100]	Match activities (total distance, high-speed running and sprinting) decreased from 0 to 15 min of the first half to the start of the second half (46–60 min) in soccer players and referees. A decrement in the technical performance of referees expressed as the distance from the ball was noted. Distance from fouls and the number of decisions were stable	Match activities of soccer players and referees were comparable. It was assumed that a slower tempo of the match caused reduced match activities during the initial phase of the second half
Weston et al. [106]	Variability on match to match was observed for high-speed running distance, recovery time, percentage of explosive sprints and the total number of sprints and fouls. The match-to-match variability was reduced for total distance covered, top sprinting speed and distance from fouls and the ball. Age and referee experience did not influence match-to-match variation	Given the greater variability between matches, future studies must include larger samples. In parallel, conditioning training should be adequate in order to prepare older referees for the demands of the game
Weston et al. [116]	Soccer referees covered 11.3 km, while players accounted for 10.8 km. Regarding the mean total distance covered, high-speed running and sprinting were comparable among soccer players and referees. Considering the entire season, total distance and sprinting were similar between players and referees	Activities of soccer players were related to referees. Soccer referees fit the needs of the game
Barbero-Álvarez et al. [97]	Field referees covered, on average, 10.2 km, while assistant referees covered 5.8 km. Regarding the frequencies of actions, no differences were noted between referees and assistant referees; however, the distance covered in accelerations was superior for field referees. Field referees tended to decrease the number of accelerations from the first to the second half, while no significant differences were found among assistant referees. heart rate, Mean expressed as the percentage of maximal heart rate, was 85.6% and 75.3% for referees and assistant referees, respectively. Curiously, the distance covered by field referees correlated with the maximal rate percentage. The effective index decreased significantly in the first half and the second half in referees. The decrement in assistant referees was noted in the second half	Acceleration activities should be part of soccer referee training programmes. The effective index needs to be considered for examining changes in performance
Mallo et al. [117]	The errors of referee and assistant referees were, on average, 14% and 13%, respectively. Among referees, the lowest percentage of error occurred in the central area of the field. The angle of vision, particularly 46 and 60o, is a crucial variable in decreasing the percentage of errors. Otherwise, the distance from the offside line was unrelated to the offside decisions. The highest error percentage tended to occur towards the end of the matches	Perceptual-cognitive demands of the game should be training among assistant referees. Additionally, the performance of assistant referees should be expressed as a percentage of successful decisions
Costa et al. [118]	No differences in the distance covered in the first (5.2 km) and second half (5.2 km) were noted. Most of the time was covered at ≥ 80% of the maximal heart rate. Subjective (rate of perceived effort) and objective (Edwards method) were associated. Significant correlations between the rate of perceived effort and distance covered at 90–100% of maximal heart rate and maximal speed were noted	GPS and heart rate monitors are helpful tools for monitoring training load. The current study highlights the need for high-intensity training programs for referees, and the rate of perceived effort is a valuable tool to control the internal load
Barberó-Alvarez et al. [119]	The present study defined repeated sprint sequences (3 consecutive accelerations—> 1.5 m s^−2^—interspersed with a maximum of 45 s of recovery) and repeated sprint ability (3 consecutive sprints—> 18 km h^−1^—interspersed with a maximum of 45 s of recovery). Note that referees and assistant referees spent 37% and 20% of the total distance in accelerations, respectively. From the first to the second half, referees decreased repeated acceleration activities	This study clarified the concepts of sprint and acceleration. Hence, acceleration testing should be part of referee programs
Gomez-Carmona et al. [120]	The current study analysed one main referee and five assistant referees in 4 matches. The referee had, on average, 179 beats per minute, corresponding to 68% of the maximal heart rate. 56% of the live time was spent at 85–95% of maximal heart rate. On the other hand, the assistant referee spent most of the time between 85 and 95% of the maximum heart rate—the mean heart rate was 139 beats per minute. Distances covered differed between referee (~ 10 km) and assistant referees (~ 6 km), as well, on match activities. Overall, errors tended to occur between 85 and 95% of maximal heart rate, and a higher percentage of error was noted on the right side of the soccer field (79%)	The authors did not present practical applications and compare the results of this study with the literature
Castillo et al. [121]	Differences between referees and assistant referees were found on match load. More specifically, referees reported higher values of internal and external loads. Regarding internal load, differentiated ratings of perceived respiratory and leg muscle were noted, with referees reporting higher values than assistant referees. The association between internal and external loads was not evident	Referees and assistant referees need to be involved in sessions with different characteristics. The present study highlights the importance of controlling internal and external loads
Dolanski et al. [122]	Referees ran 7.75 km, while assistant referees covered 4.40 km. On average, mean heart rate, maximum heart rate and mean velocity was significantly higher in referees than in assistant referees. Maximum velocity was higher in assistant referees	Referees and assistant referees require specific preparation
Fernández Elías [123]	Referees covered approximately 8.7 km. No differences were noted among distances and heart rates measured during the first and second half. Maximum velocities attained were 26.0 and 26.5 km h^−1^ in the first and second half, respectively	Strength and aerobic sessions are essential to referees
Castillo et al. [102]	In general, referees that participated in best team matches covered more distance walking (velocity defined as < 3.6 km h^−1^) and performed more accelerations and decelerations than referees who participated in lower team matches. Considering internal load parameters, referees who participated in mixed matches reported lower values of maximal heart rate. The same participants reported higher respiratory and muscular perceived match load during the second half	Short-term actions need to be considered in training programs that involve referees from higher competitive levels. The rates of perceived match load in the second half suggested the onset of fatigue
Joo and Jee [99]	Comparable match intensities (distance covered, jogging, running and sprinting) were noted among referees and players. The number of fouls, the distance between fouls and referee positioning, and foul errors were significantly higher after the 75-min. The frequency of errors tended to be higher at the locations farther away from the assistant referees	Training sessions should include perceptual and cognitive tasks to improve foul decisions
Ozaeta et al. [124]	During the second half, lower values of mean power, mean speed, mean cadence and mean and peak heart were reported among referees. They presented lower mean and peak heart rate values during the second half. However, the analysis by 15-min intervals revealed that referees covered more distance 75–90 min and performed higher mean power. Of interest, the mean heart value tended to decrease in the second half and peak power, mean velocity, mean cadence and mean vertical oscillation was higher during the first 15 min of the match	This study demonstrated that match load decreased during the game related to neuromuscular fatigue. However, during the last 15-min match load tended to increase due to team strategies
Castillo-Rodriguez et al. [103]	Physiological responses, determined by heart rate parameters and rate of perceived exertion, were higher in national referees. Differences between groups were also noted for match activities. For example, national referees covered greater distances and spent more time > 13.0 km h^−1^. Before the game, national soccer referees showed higher self-esteem and self-confidence values and presented lower cognitive and somatic anxiety values than non-national referees. A negative relationship between self-confidence and physical parameters was found. In addition, self-esteem was positively related to high-intensity activities. Finally, age was explained, and psychological responses explained 20–7.7% of referee categories	Prior to the games, national referees had better psychological responses. Physiological programs to improve the rates of chronic anxiety, somatic anxiety, self-confidence and self-esteem must be considered, especially among referees of lower categories
Moreno-Perez et al. [105]	Match congestion impacted match activities—running speed at 21–24 km h^−1^, > 24 km h^−1^, frequency of sprints and distance covered at sprint decreased moderately. Although other parameters decreased during match congestion, an interaction between congestions and halves was not found	The present study suggested specific conditioning and recovery programs during match congestion. The current also has implications for referee appointments
Martínez-Torremocha et al. [104]	Comparisons between first and second-division referees noted significant differences in mean heart rate (first division: 146 beats per minute; second division: 153 beats per minute and rate of perceived effort (first division: 7.22; second division. 7.82). Distance covered was comparable between first and second-division referees; however, different match activities were noted. Contrasting match activities by competitive level and considering the first and second halves, it was found that second-division referees showed a decrement in maximum acceleration, sprint distance per minute, maximum velocity and mean heart rate	Match characteristics and demands were different in the two different divisions. Consequently, training programs should be adapted according to the competitive levels

#### Physical Testing

The validity of field protocols was tested by comparing physical tests with internal or external match load (Table 2). Although many physical tests have been adopted among soccer referees (Additional file 4: Table S4), the relationship with match performance is inconclusive. The application of linear sprint protocols showed trivial associations with high-intensity actions during official matches [35]. Among Spanish referees from the 3rd Division, the maximum velocity derived from a straight-line sprint test (3 × 30 m; mean value: 29.5 km h^−1^) was higher than the maximum velocity recorded in the matches (referees: 23.9 km h^−1^; assistant referees: 25.7 km h^−1^) [38]. The best sprint from the 6 × 40-m sprint test was strongly associated with high-intensity match activities (i.e. high-intensity running and sprinting distance). The fastest sprint explained 58% and 56% of variance in high-intensity running and sprinting distance, respectively. The mean time from the 6 × 40-m sprint test was also largely associated (*r *=  − 0.77) with high-intensity activities [37]. Inconsistent results were verified in 28 participants (9 referees and 19 assistant referees from Norway). Repeated sprint ability protocol (mean sprint time for 5 × 30 m) was correlated with high-intensity running in assistant referees. However, no associations were noted for 6 × 40-m mean sprint times and match performance in soccer referees [39].

**Table 2:** Studies focused on testing and interventions

Study	Results/main findings	Practical applications
*Load × field tests*		
Castagna and D’Ottavio [11]	Referees covered approximately 11.6 km per game (varied between 10 and 12 km). A significant relationship between maximal oxygen uptake and total distance covered was noted. Additionally, negative correlations between maximal oxygen uptake and time standing were obtained. Overall, maximal oxygen uptake impacts the total distance covered and match intensities	Aerobic power combined with match skills should be the primary purpose of soccer referees training
Krustrup and Bangsbo [34]	The total high-intensity activities were related to the distance covered in the YOYO protocol	YOYO is a valid alternative to test match performance
Castagna et al. [35]	Velocities at 2 and 4 mmol l^−1^, obtained from a field protocol, were 10.9 km.h^−1^ and 13.6 km h^−1^, respectively. Velocity attained at 4 mmol^−1^ was associated with the total distance covered during the soccer game. However, it was not related to high-intensity activities	The velocity at the blood lactate threshold of 4 mmol l^−1^ helps to develop training programmes among referees
Castagna et al. [12]	Associations between field protocols (50-m sprint, 200 sprint and 12-min run) and match activities were poor and moderate. In fact, 12-min run test had a positive and moderate association with distance covered at high intensity	The 12-min run test should be used to monitor aerobic fitness. Given the level of referees included in this study, a benchmark of 3 km was defined as an advisable aerobic fitness level
Krustrup et al. [13]	Significant correlations between high-intensity running and repeated sprint test, maximal oxygen uptake and 50-m sprint were found. Meantime, no associations were found between 12 min of running or treadmill protocol with high-intensity activities during the game. Significant relationships between the distance to the offside line and time to complete three sprints after the game and the best trial of the 50-m sprint were obtained	(see match indicators)
Tessitore et al. [14]	On average, the maximal oxygen uptake was 51.8 ml kg^−1^ min^−1^, and the mean heart rate was 194 bpm, corresponding to 98% of the maximal heart rate. High-intensity activities (86–95% of maximal heart rate predominantly occurred during the first half). Blood lactate tended to be predominantly elevated after the first half. The performance regarding jump ability showed no significant differences before the match, after the first and second halves	Referees had lower values of maximal oxygen uptake compared to soccer players, probably because they have lower training volume. Referees with adequate fitness levels can maintain performance during the match
Mallo et al. [36]	The reference values for the 12-min running test and the 50-m sprinting were 2962 m and 6.89 s, respectively. Note, no significant association were found between field protocols and match performance	Adequate fitness tests, such as YOYO intermittent interval test and 30- to 40-m sprinting, need future consideration
Mallo et al. [22]	The protocol adopted to evaluate physical capacity (6 × 40 m) did not significantly predict match activities	Protocols adopted by FIFA were poorly related to match analysis parameters
Weston et al. [37]	Associations between FIFA protocols and match parameters were found—the fastest 40-m sprint and distance covered were related to high-intensity running and sprinting distance. In addition, the mean time 6 × 40-m sprint test was associated with the total distance covered, high-intensity running and sprinting distance. On average, the fastest 40-m sprint time was 6.92 s, and the mean of the respective protocol was 5.71 s. Referees covered, on average, 11.5 km	The 6 × 40-m sprint test and, in particular, the fastest sprint is appropriate to assess the physical fitness of soccer referees
Castillo et al. [38]	Referees and assistant referees did not differ on the straight-line sprint test (SLST); however, significant differences were noted in the maximum speeds during official matches. Additionally, among referees and assistant referees, the maximum speed attained in the game was significantly lower than the maximum speed registered in the SLST	Velocities of SLST (3 sprints × 30 m) are not comparable with velocities obtained during the games thus, other protocols need to be validated according to match activities
Castillo et al. [51]	After the match, referees and assistant referees tended to increase blood lactate and sprint time, while decrement in jumping performance was unclear	Match-related fatigue was evident in referees and assistant referees
Risser et al. [39]	The change of direction ability test was not related to high-intensity running activity and accelerations among assistant referees. In opposition, 5 × 30-m sprint test was associated with distance in high-intensity running. Match activities and 6 × 40-m sprint test were not associated among referees	Negligible associations between field protocols and match activities were found, which may is explained by the large variability of match-to-match activities. Match performance is associated with other variables rather than field protocols
Castillo et al. [40]	Referees and assistant referees did not differ in physical performance (linear straight sprint test, modified agility t test free and YOYO distance). Linear straight sprint test and distance covered in the YOYO protocol were related to high-speed and high-intensity match activities (coefficients of correlation ranged from moderate to large) among referees. However, linear straight sprint test and high-speed running were associated with assistant referees. Linear straight sprint test was correlated with very high-intensity accelerations and decelerations. Accelerations and very high-intensity decelerations were related to the distance covered in YOYO	External match load was higher in referees than assistant referees, but they had comparable physical conditions. The authors highlighted that training strategies are equivalent for referees and assistant referees. Training sessions of referees should include sprint ability exercises
Preissler et al. [41]	Maximal and mean heart rates during official matches were significantly higher than on field tests. On the other hand, speed parameters (maximum and mean) were higher on physical tests. As expected, the distance covered was also higher in the matches. The percentage of time (70–80% of maximal heart rate) was longer in the match than in the test, while an opposite tendency was obtained for 90–100% of maximal heart rate. Speed zone below 13 km h^−1^ were longer in the match, while speeding zones above 13 km h^−1^ (13–18 km h^−1^; 18–24 km h^−1^; > 24 km h^−1^ were longer in the match	Physical tests and match performance require a specific stimulus of training. Before the test, referees are advised to focus their training in anaerobic stimulus, while during the season aerobic training should complement training programs
*Physical testing (age variation, competitive level, data quality)*		
Castagna et al. [45]	12 m run test did not discriminate referees by competitive level. Meantime, top-level referees covered more distance in YOYO protocol than medium and low-level referees. Top referees also show low blood lactate levels determined immediately at the end of the maximal intermittent test	YOYO protocol should be used to select soccer referees
Castagna et al. [15]	A negative relationship between CMJ, 12-min running and age was noted. Although maximal oxygen uptake was higher in the younger group, older referees tend to attain similar velocity at 4 mmol L^−1^	The present study presented reference values according to age groups: 3030 m (30 years) to 2700 m (42 years); performed 6.90 s and 28 s over 50-m and 200-m sprint tests, Respectively. Referees should also perform VO_2max_ near 50 ml kg^−1^ min^−1^
Casajus and Castagna [42]	Age-associated variation in maximal oxygen uptake, 12-min running test and 200 m were not noted, as well as in body size and composition. Meantime, maximal heart rate and power at the ventilatory threshold were significantly higher in younger referees compared to older peers	Training programmes should include aerobic and anaerobic activities. In order to maintain acceptable aerobic performance, older referees need to assess body mass and composition
Caballero et al. [17]	Two field tests were used: 6 × 40 m and 2 km running. In the first test, the mean time was 5.53 s, while the best sprint was, on average, 4.99 s. 2 km was completed in 7 min and 43 s	Performance expressed by 2 km showed a lower association with VO_2max_. Thus, it is not recommended to assess physical fitness
Castagna et al. [46]	The best time of CODA (change of direction ability test) was 9.81 and 9.66 s for the first and second visions, respectively—no statistical differences were noted between groups. No differences were not noted for the mean performance. The protocol did not discriminate competitive level. The intra-class correlation coefficient and technical error of measurement were 0.90 and 0.18, respectively	Percentiles for the current test estimated a cut-off value of ≤ 9.67 s as acceptable. The protocol was reliable and should be considered in test protocols
Castagna et al. [8]	The intermittent endurance test was reproducible, discriminating assistant referees from different competitive levels, and was related to YOYO and VO2_max_	The intermittent endurance test seems to be a proper protocol for assessing the intermittent endurance capacity among assistant referees
Castillo et al. [43]	Field protocols did not discriminate referees and assistant references, nor national and provincial referees. Younger referees (≤ 35 years) were faster in sprint and agility protocols and, in addition, covered more distance in the YOYO test. Agility and linear velocity tests were related	Linear sprint tests did not discriminate referees according to the competitive level. Given the age-associated variation in field protocols, training programs should be focused on maintaining agility, acceleration and aerobic capacity in referees, particularly at older ages
Yanci et al. [125]	Modified Agility Test time was higher in regional referees, while differences between national and regional referees were not found for linear sprint protocols (5-m and 15-m sprint tests)	Modified Agility Test can be used to discriminate referees by competitive level
Castillo et al. [18]	During the preparatory period, referees and assistant referees tend to decrease sprint performance assessed by 20 and 30-m tests. On the other hand, referees increased 13.11% the distance performed on the YOYO protocol, while assistant referees decreased 3.36% the distance on YOYO test	Acceleration exercises and lower limb power training need to be implemented among referees during the off-season. In parallel, aerobic sessions during the off-season among assistant referees are needed
Gianturco et al. [47]	The foot posture index (assessed by a questionnaire) was associated with the performance on the YOYO test	The foot posture index, a reasonable indicator of function, needs to be considered in training sessions
Sanchez-Garcia et al. [48]	The total distance covered on the YOYO protocol was only moderately associated with the maximum velocity obtained in the 40-m sprint test. The maximum velocity obtained in the 40-m sprint test and YOYO distance were unrelated to the time to cover 40 m	YOYO protocol seems to have limitations in assessing high velocities
Meckel et al. [44]	Second-division referees were significantly faster in ideal and accumulative sprint times than first-division referees at the beginning of the season (baseline) and mid-season assessment. In addition, second-division referees improved their performance from the baseline to the mid-season. Among referees, age was significantly related to ideal sprint (*r *= 0.63) and total sprint time (*r *= 0.66)	The highest level of soccer referees may be explained by something other parameters than physical performance. Experienced referees adopted skills (e.g. positioning, anticipation, decision, running economy) that compensate for physical disadvantages. Younger referees should focus their training plans on tactical drills, while older referees need to focus their training plans on physical capacities
Muscella et al. [16]	Sprint time (40-m test) tended to decrease across the season, while the distance in the YOYO protocol increased. Age was negatively associated with changes on the 40-m sprint test and distance covered on the YOYO protocol. Younger referees showed a higher ranking than intermediate (21–29 years) and older (30–45 years) referees	This study showed that young referees attained better sprint performance and were ranked top
Romano et al. [126]	The 6 × 40-m sprint (best sprint) was positively associated with time to complete an agility test. In contrast, the relationship between the 6 × 40-m sprint (best sprint) and with YOYO protocol was significant but negative. Moderate associations between hand-grip strength and YOYO test were obtained	Hand-grip test and Illinois agility tests are valid alternatives to monitor fitness among referees
Muscella et al. [96]	Across the season, players substantially improved the 40-m sprint test and distance covered in the YOYO test. Changes in cortisol and testosterone were particularly noted from the beginning of the season to the first moment of assessment (8 weeks from baseline) and then systematically decreased until the end of the season (June), attaining the baseline levels. On the other hand, testosterone increased eight weeks from T0 until mid-season and then decreased towards initial values. At moment 1, cortisol and testosterone were related to distance covered on YOYO. At the moment 2, only testosterone was associated with distance covered on YOYO protocol	Referee training promotes physiological adaptations that can be examined by changes in testosterone and cortisol levels. These parameters can be indicators of training adaptations and overtraining
Castagna et al. [49]	YOYO distance covered tended to increase across the 15 months of observation. On the other hand, the time to complete the linear sprint ability test, 5 × 30 m (best sprint) and change of direction protocol increased from the baseline. A very large correlation was noted between the linear sprint ability test and 5 × 30 m (*r *= 0.89). Intra-class correlation coefficients for the protocols ranged from good to very large.	Given the association between 5 × 30 m (best sprint) and repeated sprint ability and the lower variability in best sprint obtained from 5 × 30 m, a single maximal sprint in of referees may provide the same level of information as repeated sprint ability protocol. Repeated sprint ability and linear sprint tests consider two different types of neuromuscular performance
*Interventions*		
Krustrup and Bangsbo [34]	The intermittent exercise intervention had an impact on the high-intensity distance covered (after: 2.06 ± 0.13 km; before 1.69 ± 0.08 km) and heart rate (after: 159 ± 1 beat per minute; before:164 ± 2 beats per minute)	A high-intensity intermittent protocol (long-duration running intervals: 4 × 4 min or 8 × 2 min; short-duration running intervals: 16 × 1 min or 24 × 30 s) improved performance during the game
Weston et al. [54]	No differences between track and pitch in the mean percentage of maximal rate were noted. However, the maximal heart rate was significantly higher during the track training sessions. After applying high-intensity intermittent training, the mean distance covered in the YOYO protocol increased from November until March	The present study shows practical training sessions for elite referees (see p.56) to improve fitness levels
Boullosa et al. [50]	Moderate to large correlations were found between resting heart rate variability and the rest-to-match day. YOYO test was not correlated with heart rate variability	Cardiac autonomic control decreased in the 5 h before the match until 10 h after the match. In parallel, referees with greater variability on a rest day may tolerate the stress before, during and after exercise. Heart rate variability should be used to assess stress (physical and physiological) and recovery in soccer referees
Castillo et al. [51]	After the game, decrements of 15-m and 30-m sprint tests were noted among referees and assistant referees. After the game, referees and assistant referees increased blood lactate. Differences between groups were found	The current study suggested that soccer matches caused fatigue. Sprint exercises should be part of training sessions
Castagna et al. [127]	The training rate of perceived effort showed no differences immediately after training. Thirty minutes post-training, 7 h post-training and 20 h post-training	The present study showed no significant differences in recall timing on post-exercise RPE when athletes are familiarized with the scale
Castillo et al. [52]	Soccer referees improved jumping performance (different variables were analysed) after the matches	Soccer matches did not cause decrements in vertical jump performance, indicating that neuromuscular fatigue caused by the matches is insufficient to modify jump variables
Yanaoka et al. [58]	The distance covered on the YOYO protocol was significantly longer in the re-warm-up group than in the control trial. After the intervention, no trial × time effect was noted on plasma glucose, fat-free serum acids, serum triglycerides, blood lactate and creatine kinase. As expected, the perceived effort rate was higher in the re-warm-up group	Re-warm up is an effective method to improve performance, although the results of the current study were limited to the YOYO protocol. The present study proposes the following intermittent halftime protocol: 2.15 min of seating rest and 2.15 min of running at 70% of maximal heart rate
Maslenniov et al. [55]	After applying the experimental protocol (p.10, Fig. 2 in the original manuscript), negative classifications decreased in the experimental and control groups. The total distance covered was higher in the experimental group	The authors suggested including physical tests in the annual cycle of soccer referees
Muniroglu et al. [56]	The flag had a negative impact on sprint protocols (5, 10, 20 and 30 m) among assistant referees. The initial position of referees (straight or lateral) did not influence the performance in field protocols. Training with a flag promoted substantial performance improvements	Assistant referees should include a flag during training sessions to improve speed
Baydemir et al. [57]	After a 16-week intervention of high-intensity interval training and sprint sessions, referees significantly decreased body mass and improved the performance on FIFA protocols (40 × 75 m; 6 × 40 m) and the Cooper test	The current study showed a training cycle positively impacting FIFA (6 × 40 m) and Cooper protocols among amateur referees
Fernandez-Ruiz et al. [53]	Soccer matches did not influence isometric knee flexion strength in the dominant limb but the isometric body strength of the non-dominant limb among referees. Differences were noted in assistant referees. Differences after the match were negligible in the range of motion of hip flexion in dominant and non-dominant limbs in both groups	Strength programs need to be developed for referees in order to reduce differences in isometric hamstring strength after the match

Contradictory results regarding the associations of the match performance with distance covered in long-term field protocols were also obtained. The YOYO intermittent recovery protocol was associated with high-intensity match activities in referees from different competitive levels [33, 34, 40]. Non-significant associations between the 12-min running test and total distance covered (*r *= 0.24) or time spent in high intensity (*r *= 0.35) in assistant referees were found [36]. Moreover, the 12-min running test did not discriminate referees of different competitive levels but, top-level referees covered 514 m more and 602 m more during YOYO protocol than those in third or fourth divisions, respectively [45]. The change of direction ability test was reproducible in referees [46] and also differentiated national referees from regional referees [125]. The intermittent endurance protocol was reproducible for assistant referees [8].

The effect of age on physical performance is not conclusive (Table 2). Studies found a negative relationship between age and the 12-min running test, countermovement jump [15], 40-m sprint test and distance covered on the YOYO protocol [16]. In contrast with the previous results, two papers reported that younger and older referees attained similar velocities at 4 mmol L^−1^ of blood lactate [15] and an age-associated variation was not found in maximal oxygen uptake, the 12-min running test or the 200-m sprint test [42].

High-intensity intermittent protocols improved physical performance in soccer referees [34, 54, 57]. In addition, a re-warm up intervention demonstrated considerable improvements in the endurance performance by YOYO test [58]. Strength, jump and velocity performance were reduced after a soccer match [51, 53].

#### Nutrition

**Fig. 3:**
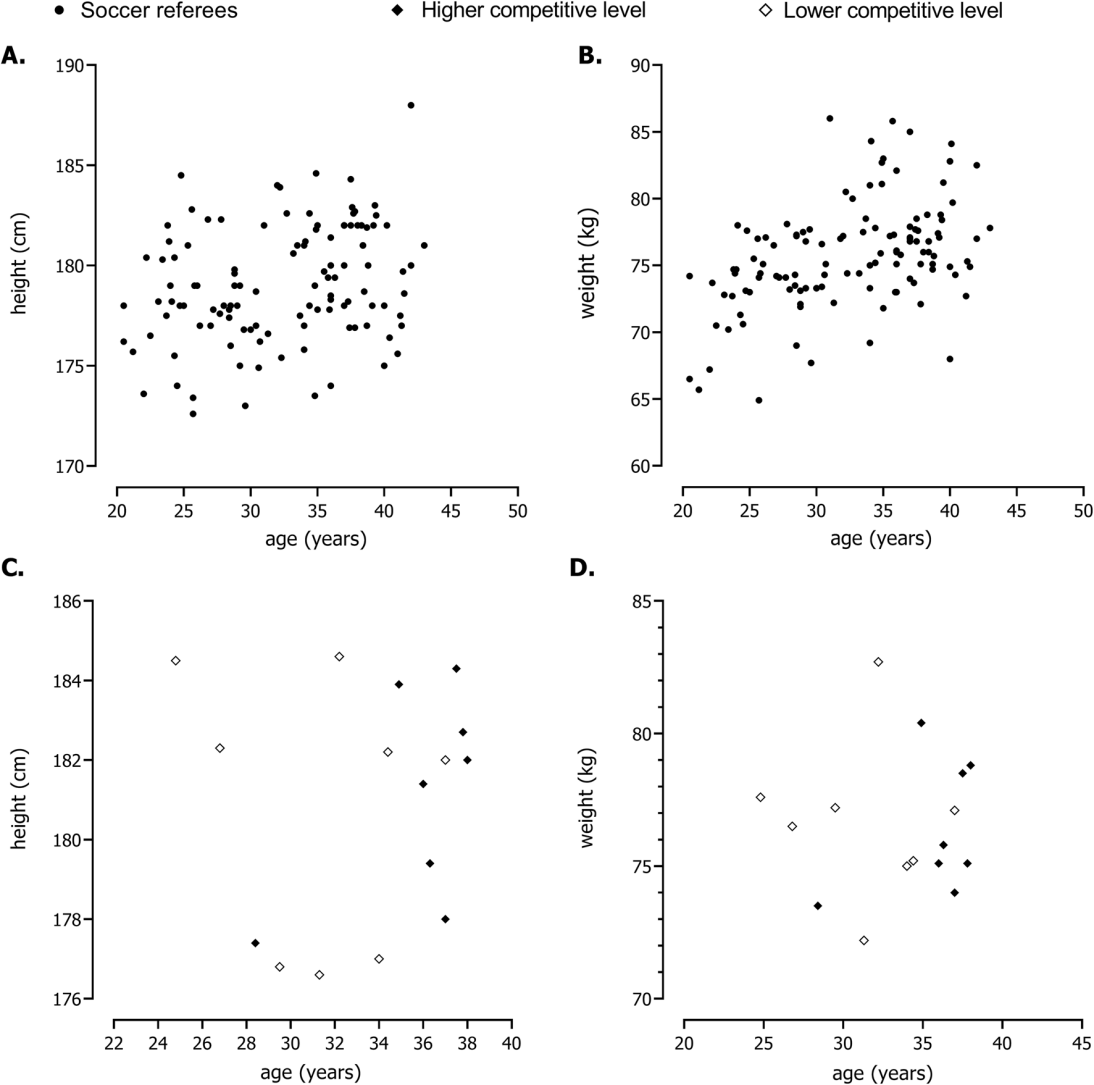
Heights (**a**) and weights (**b**) of soccer referees plotted against age; heights (**c**); and weights (**d**) by competitive level

**Fig. 4:**
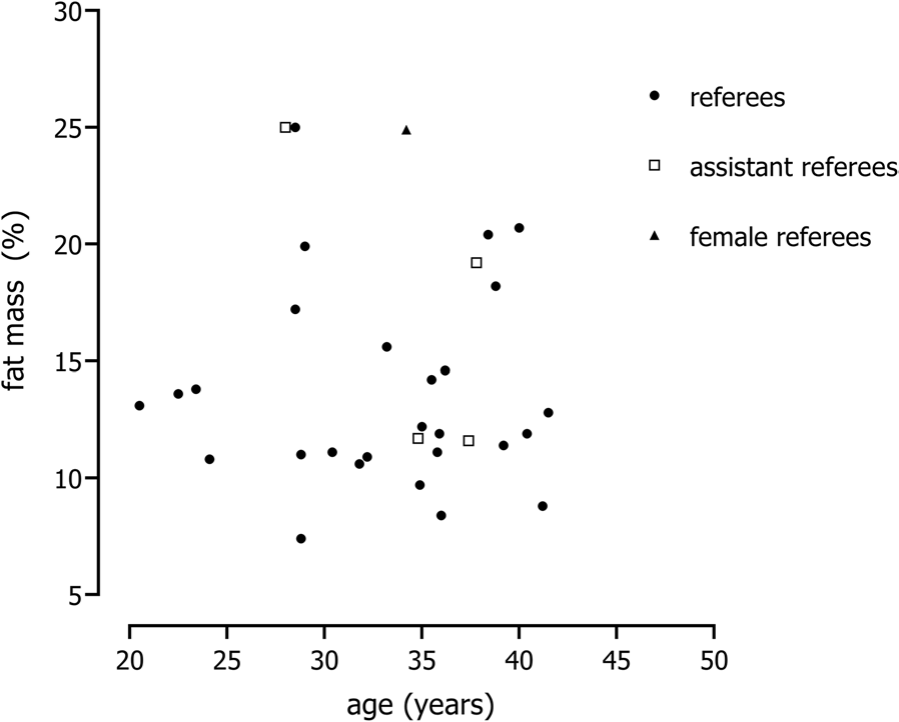
Fat mass percentage estimated from different methodologies plotted against age in soccer referees

This topic included the articles focused on body size, body composition and nutrition. Height was associated with competitive level and authority [59, 60] and match statistics (i.e. the number of yellow cards, red cards and penalties given per match) [24]. Although few studies reported fat mass, an overall conclusion that emerged from the included studies was the inverse relationship between age and fat mass percentage [61–63]. Brazilian referees aged 20–30 years had, on average, 16.9% body fat percentage, while older groups tended to present values of fat mass percentage around 19% [61]. Lower values of fat mass percentage obtained by bioimpedance analysis were found for Spanish referees (mean: 7.8%), and younger officials presented less fatness than their peers [62]. Referees tended to present lower values of fat mass compared with assistant referees [62, 64, 67]. Based on dual-energy X-ray absorptiometry, fat mass assessment for referees and assistance referees was 15.6% and 17.8%, respectively [64]. Mean heights and weights obtained from the studies included in the current review were plotted against age. The data suggested a reasonable degree of heterogeneity in the distributions of height and weight among referees of different age groups (Fig. 3a and b). Using of height and weight to discriminate between soccer referees by competitive level was not evident [44–46, 87, 101, 104, 125] (Fig. 1c and d). As shown in Fig. 4, the majority of data points referring to fat mass percentages were below 15% (21 of 34 data points).

Total energy intake was comparable in three studies (Table 3); however, macronutrient intake did not meet the nutritional recommendations [72–74]. Only three studies estimated the energy expenditure during training sessions [71] and matches [61, 68].

**Table 3:** Studies focused on nutrition topic

Study	Results/main findings	Practical applications
*Energy expenditure, energy and nutritional intake*		
da Silva et al. [68]	Energy expenditure, estimated by indirect equations using the total time in each activity, revealed a total energy expenditure during the match of 734 kcal (first half: 374 kcal; second half: 359 kcal)	Nutritional strategies should be adapted to referee activities
Silva et al. [69]	The ingestion of a pre-determined volume of carbohydrates reduced the sweat rate to 0.72 ± 0.12 l h^−1^ compared to ad libitum (1.16 l min^−1^). In addition, the time spent in low-intensity activities was also reduced by 69.9% with a carbohydrate solution	Carbohydrate rehydration is an effective solution to decrease the time spent on low-intensity activities
Teixeira et al. [72]	On average, the daily energy intake was 2819 kcal day^−1^ or 36 kcal day^−1^. Regarding the macronutrient intake, soccer referees did not fit the recommendations of carbohydrates (4.1 g kg^−1^). On the other hand, the average intake of protein is near the upper limit of guidelines (1.7 g kg^−1^). Saturated fat exceeded the recommendation and polyunsaturated fats were below the recommendations. Folate, vitamin A, vitamin C, magnesium and calcium intakes did not meet the recommendations. 78% of participants exceeded the recommendations of sodium intake	The intake of carbohydrates could compromise the preparation and recovery from training sessions and matches. Therefore, referees need nutritional advice
Renon and Collado [73]	The daily energy intake was, on average, 2409 kcal day^−1^. The intake of carbohydrates was low, while the protein intake was high. No differences in carbohydrate intake were found according to the type of day (rest, training and match), although referees tended to ingest more carbohydrates on match days. The intake of vitamins B1, B2, B6, B12 and minerals (calcium, magnesium, iron) exceeded the guidelines. On the other hand, magnesium intake met the recommendations. No differences in minerals and fibre were found by type of day	Overall, soccer referees ingested limited carbohydrates, while protein intake exceeded the recommendations. The periodization of nutrients needs to be adjusted appropriately for the type of day
Metz et al. [74]	Energy intake was higher during the game day compared to the day off (game day: 2782 kcal; day off 2270 kcal). Considering the two conditions (game day vs day off), differences between macronutrients were significant for carbohydrates, while negligible differences in protein and fat intake were found. Carbohydrate intake tends to increase during breakfast and snacks and, in parallel, decrease during dinner. The stress level before each meal tended to be higher on match day	Soccer referees must adapt their intake according to match demands or recovering days
Paes and Fernandez [70]	Backward movements at velocities similar to jogging and walking showed an energy expenditure of less than 9 kcal. Energy expenditure estimated by mathematical equations overestimated the observed values	Backward movements should not be defined as high-intensity activities, and equations to predict energy expenditure have associated errors related to higher body fat levels found in referees
Malaguti et al. [71]	Using a watch, energy expended during the training sessions (referees: 997 kcal; assistant referees: 701 kcal) was lower than during the matches (referees: 1266 kcal; assistant referees: 994 kcal). Referees from a lower competitive level tended to consume more dietary supplements following a friendly suggestion. 46% of international referees reported that they follow the advice of an expert	Referees did not have adequate knowledge about supplements and their impact on soccer. With this in mind, official federations should promote educational lessons about nutrition
Gacek et al. [75]	Referee experience was positively associated with the frequency of fruit, dairy products, nuts and alcohol consumption. On the other hand, carbohydrate drinks, sea fish and cereals were negatively related to experience. The self-efficacy questionnaire was associated with the frequency of consumption of dairy products with fat content, eggs and mineral water	Overall, the nutritional choices of soccer referees tended to improve with age and experience
Mascherini et al. [76]	Soccer referees ingested 3.1 g kg^−1^, 1.3 g kg^−1^ and 1.1 g kg^−1^ of carbohydrates, fat and protein, respectively. Regarding micronutrients, soccer referees ingested 661 mg day^−1^, 2929 mg day^−1^, 794 mg day^−1^, 10.3 mg day^−1^, 21.2 μg day^−1^, 0.3 μg day^−1^, 381 μg day^−1^ of calcium, potassium, zinc, magnesium, iodine, vitamin B12 and vitamin B9, respectively	Regarding the nutritional intake of soccer, referees did not follow the guidelines proposed for soccer players
*Body size and composition*		
Krustrup et al. [13]	Assistant referees loss on average 0.8 kg, which corresponds to 1.0% of body mass. They ingested 0.36 l of fluids thereby, estimated sweat rate was 1.17 l	(see Table 2)
Silva et al. [61]	The mean age of present sample was 33.7 years. Mean value of fat mass percentage was 18.5%. Younger referees (20.0–29.9 years) showed lower values of fat mass percentage in comparison to the other age-groups (30–39.9 years; ≥ 40 years). No differences were found between national and regional referees	Referees presented higher values of fat mass than soccer players and, consequently, nutritional interventions should be implemented
Stulp et al. [59]	Referees were on average taller 4 cm than assistant referees. Height was associated with authority and effectiveness on the match. Taller players compared shorter referees mark less fouls	Taller referees maintained the control of the match once mark less fouls
Casajus et al. [62]	Referees had lower values of fat mass percentage than assistant referees. Age comparisons indicated that younger referees had lower fat mass percentage than older referees	Values of fat mass percentage were within the normal range of healthy population. Body composition assessment should be part of FIFA fitness evaluation
Casajus et al. [63]	Body fat was significantly lower in referees (1st, 2nd and 2nb B division) than assistant referees (1st and 2nd divisions). Comparisons between age groups showed that older referees had, on average, higher values of fat mass percentage than younger referees. A trend towards the stabilization of fat mass across 1-year among referees was noted—the values ranged 8–14%	The stabilization of fat mass percentage between 8 and 14% is particular important to achieve the standards required to be a referee. Monitorization of fat mass should be considered across the season
Bozdogan et al. [77]	Among referees, body fat had impact on YOYO distance covered and repeated sprint ability test (total time). Among assistant referees, body fat was associated with repeated sprint ability test	Given the negative impact of fat mass on running performance, appropriate training programs and nutrition interventions should be considered
Banda et al. [60]	Referees were 4.5 cm than assistant referees. Differences by between competitive level suggested that FIFA referees had, on average, lower values of fat mass percentage and sum of six skinfolds in comparison to ZIFA referees	Age is an important parameter to attain refereeing elite and body composition should be monitored, particularly at older ages
Casajus et al. [64]	Variables of body composition extracted from bioimpedance and dual-energy X-ray absorptiometry showed significant differences. Assistant referees had higher values of fat mass than referees (assistant referees: 17.8% with dual-energy X-ray absorptiometry, 13.9% with bioimpedance; referees: 15.6% with dual-energy X-ray absorptiometry; 11.7 with bioimpedance). In general, and considering all sample (referees and assistant referees), bioimpedance underestimated 3.87% fat mass and overestimated 3.56 kg fat-free mass compared with dual-energy X-ray absorptiometry	Optimal body composition is required to promote referees to elite level. In this context, general equations based on bioimpedance should not be used among soccer referees. In contrast, specific equations need to be developed
Castillo et al. [40]	10-week competitive period at end of the season noted a significant decrement on skinfold thickness and, in opposition, lean body mass increased 1.1 kg	Intense competition at the end of the season caused changes in skinfolds, estimated fat mass and lean body mass
McCarrick et al. [24]	The present study suggested that height was related to the number of yellow, red cards and penalties registered by match in lower competitive leagues. Contrasting results were obtained to higher competitive leagues	The impact of height on punitive actions is dependent of the competitive context
Petri et al. [66]	Correlation coefficients between fat mass percentage derived from dual-energy X-ray absorptiometry and anthropometry varied between moderate to very large (the only exception was the sum of anterior and medial calf). The current study presented a skinfold model to estimate fat mass percentage derived from dual-energy X-ray absorptiometry: %FM = 8.386 + (0.478 × iliac crest skinfold) + (0.395 × abdominal skinfold (*r* = 0.781; *R*^2^ = 0.610; SEE = 2.62%)	National and international referees should assess body composition based on the proposed anthropometric equation
Lopez-Garcia et al. [67]	Assistant referees presented, on average, higher body fat mass percentage than referees based on anthropometry (referees: 21.3%; assistant referees: 22.7%) and dual-energy X-ray absorptiometry (referees: 20.9%; assistant referees: 21.5%)	Differences between groups were partially explained by the physical demands of soccer matches

#### Physiology

Different physiological variables were analysed among soccer referees—heart dimensions [17, 78–80], maximal oxygen uptake [17, 81–85] and strength [86, 87]. Two separate studies conducted in Brazil suggested that maximal oxygen uptake was lower in referees than soccer players [81, 85] (Table 4). Only one study compared aerobic fitness in male and female referees [83]. When normalized for weight, maximal oxygen uptake was significantly lower in females (48.1 ml kg^−1^ min^−1^) than male referees (51.9 ml kg^−1^ min^−1^) [83]. However, it did not discriminate referees and assistant referees [84].

**Table 4:** Studies focused on physiological outputs

Study	Results/main findings	Practical applications
Galanti et al. [78]	No differences were noted between soccer and referees in the following parameters: left ventricular mass, aortic root diameter, left ventricular diastolic dimension, septum and posterior wall thickness	In general, the comparisons of this study suggested comparable heart dimensions between soccer players and referees
Caballero et al. [79]	The rest heart rate was, on average, 59 beats per minute. Linear measurements of the systolic diameter of the left ventricle, the diastolic diameter of the left ventricle and the diameter of the left atrium were 33.7 mm, 50.0 mm and 29.8 mm, respectively. In addition, the thickness of the left ventricular septum was 9.77 mm, and the posterior wall of the left ventricle was 9.47 mm. The mean diastolic volume of the left ventricle was 135 ml, and ejected volume was, on average, 47.3 ml. Estimated left ventricular mass ranged 86–349 g, and the mean value was 219 g	Echocardiography parameters indicate an increase in the cardiac chambers and average values for the thickness of the walls. Hence, the increased size of the ventricle explains the left ventricular mass
Caballero et al. [17]	Biochemical parameters were obtained for haemoglobin (14.9 g dl^−1^), iron (87.5 g dl^−1^), ferritin (85.1 g dl^−1^), transferrin (276 g dl^−1^) and red cells (4.9 μl). Linear measurements of the left ventricle were 34.8 mm, 51.2 mm, 9.8 mm and 10.1 mm for systolic diameter, diastolic diameter, posterior wall and septum thickness. Left ventricular mass was, on average, 122 g. VO_2max_ evaluated during an incremental treadmill test was, on average, 48.7 ml kg^−1^ min^−1^, while the maximal heart rate was 190 beats per minute. The mean heart rate at the second ventilatory threshold was 175 beats per minute, corresponding to 92.7% of the maximal heart rate	Blood parameters did not match with aerobic training parameters. With this in mind, the authors recommend frequent assess of haematological parameters
Silva et al. [81]	Referees were, on average, older, fatter and had lower values of VO_2max_ than soccer players. No differences were found in height and weight	FIFA battery needs to include tests to assess aerobic power. Additionally, nutrition guidelines should be developed in order to improve the athletic profile
Palmer et al. [86]	Absolute and relative torque development at ½ and ¾ of maximal voluntary contraction of the hip and thigh posterior muscles were higher in full-time referees than part-time referees. No differences between groups for peak torque and maximal power were noted	The current study shows that the isometric extension of the hip allows discriminating professional and semi-professional referees. Consequently, training programs should include exercises to increase relative torque development of the hip and thigh
Mazaheri et al. [82]	Cardiorespiratory parameters were, on average, 159 beats per minute and 59.94 ml kg^−1^ min^−1^. The physiological parameters examined in this study, VO_2max_ and forced vital capacity, were not related to the mean score assigned for each referee	Body composition and physiological parameters were not associated with referee scores. Thus, cognitive aspects, psychological factors and experience may explain performance
Castagna et al. [83]	Male and female referees differed significantly on aerobic outputs—male and female referees attained a respiratory exchange ratio of 1.20 and 1.09, respectively. Female referees VO2max (48.1 ml kg^−1^ min^−1^) was significantly lower than in males (51.9 ml kg^−1^ min^−1^), and the peak velocity on the treadmill. After scaled body mass, only one female referee presented VO_2max_ higher than the mean value of male referees	In order to promote female referees in male competitions, female referees need additional aerobic training
Coffi et al. [80]	At baseline, 51.3% of soccer referees showed normal heart geometry, 37.8% presented concentric remodelling, 8.1% exhibited eccentric hypertrophy, and 2.7% had concentric hypertrophy. After the championship, the participants who presented ventricular hypertrophy did not substantially differ in heart morphology	Structural and functional parameters of the heart were classified as usual among referees
Talovic et al. [87]	Premier league referees of Bosnia and Herzegovina presented higher values on total work than first league referees. The ratio of non-dominant knees indicated that Premier League referees had a higher ratio than first league referees (3.7%)	Asymmetry and total work discriminated referees by competitive level
Castagna et al. [84]	Maximal oxygen uptake, expressed in absolute terms, was significantly higher in referees (3.98 l min^−1^) than in assistant referees (3.64 l min^−1^). Differences between groups were attenuated when maximal oxygen uptake was expressed by body mass, as well as on maximal aerobic speed and speed to exhaustion. Differences in running efficiency were negligible between groups. The speed at the ventilatory threshold was near 14 km h^−1^. At the ventilatory threshold, heart rate, expressed as a percentage of the maximal value, was 91% in referees and assistant referees. The analysis in the present study developed cut-off values for aerobic parameters—maximal oxygen uptake (3.93 l min^−1^; 50.6 ml kg^−1^ min^−1^), peak treadmill speed (16.8 km h^−1^), the ventilatory threshold at maximal oxygen uptake (42.6 ml kg^−1^ min^−1^), blood lactate (10.8 mmol l^−1^)	The similarities in training programs may explain comparable values on aerobic parameters among referees and assistant referees. The authors suggested that aerobic training (to improve maximal oxygen uptake) should adopt short intervals (15 s to 2 min), and the heart rate should be above 95% maximal heart rate
Santos-Silva et al. [85]	Significant differences between soccer players and referees were found for age (referees: 34.8 years; players: 20.8 years), maximal oxygen uptake (referees: 54.7 ml kg^−1^ min^−1^; players: 58.8 ml kg^−1^.min^−1^) and maximal heart rate (referees: 184 beats per minute; players: 192 beats per minute). No differences were found between groups in other relevant aerobic parameters, mainly velocity at maximal oxygen uptake, the velocity at the second ventilatory threshold, percentage of maximal oxygen uptake at the second ventilator threshold and respiratory exchange ratio	Aerobic assessment should be required in international referees rather than age alone

## Discussion

The purpose of this systematic review was to synthesize the literature on match indicators, physical testing, nutrition and physiological profiling of soccer referees and assistant referees. There were no effects of age on match performance. Therefore, age may not present an acceptable indicator to determine international-level participation across soccer referees. Match performance is not similar among referees and assistant referees and it is also influenced by many factors (e.g. competitive level and competitive schedule). Consequently, match activities should not be exclusively used to corroborate physical tests. Height and weight did not discriminate referees, while 15% fat mass seems to present an acceptable value for referees.

Age is used as a criterion for international-level participation, with 25 and 23 years being the minimal age for a candidate for the annual FIFA Refereeing International List [7]. The candidates are selected by the member associations within FIFA, with the Portuguese Soccer Federation, for instance, adopting the age range of 25–37 years for referees and 31–39 years for assistant referees [128]. The upper age limits imposed by the governing bodies may require attention since the present review showed contradictory findings associated with age variation in match performance [54, 103]. Among 22 English Premier League referees followed between 2003 and 2007, younger referees (31–36 years) covered 907 m more in total distance and 266 m more in high-intensity running than older referees (43–48 years). However, differences between age groups were negligible for optimal positioning (i.e. distances from the ball and fouls) [54]. This suggests that although younger referees might cover greater overall and high-speed distance, older referees might not be disadvantaged based on the subsequent adjustments to positional sense, which might be gained through increased experience. Nevertheless, match performance is also influenced by other potential variables, such as competitive level [102–104] or competitive schedule [105]. These aspects might explain the variability between studies in match performance among referees and assistant referees.

Allowing for variation in match performance across studies, two important conclusions emerged from the present systematic review: referees and assistant referees differed on total distance covered, high-intensity actions, heart rate, type of movements and ratings of perceived effort [43, 89, 97, 112, 115, 120, 122]. Soccer referees covered higher total distance and high-intensity running [89] and also demonstrated higher ratings of perceived effort, maximum heart rate and mean heart rate than assistant referees [122]. Consequently, specific training programs should be developed for referees and assistant referees. Unfortunately, few studies have focused on training interventions among referees and assistant referees. In the present review, four intervention studies focused on referees [34, 54, 55, 57], while one study included assistant referees [56]. In the 2001–2002 season, the Belgian Football Federation introduced weekly training for referees comprising four purposes for each training session, including high intensity, speed endurance, speed training and recovery. The high-intensity intermittent training exercises performed on the pitch and track sessions were detailed in the original paper (p. 56S). After 16 weeks of intervention, high-intensity intermittent exercises improved the distance covered during the YOYO test [54]. In a separate study, high-intensity interval training and speed were effective strategies to improve the number of repetitions on 40 × 75 m FIFA protocol and distance covered on the COOPER test in 25 Turkish amateur referees [57]. Another study in assistant referees showed that 5 months of training with a linesman flag improved the sprint performance [56]. Therefore, the current lack of synergy between referees and assistant referees in relation to match demands and training practices means that both of these populations should be separately considered. Interventional studies in soccer referees and assistant referees, more specifically the training programs applied by the national organizations [128, 129], need future consideration.

Evidence is lacking as to whether the physical tests are aligned with the demands of the game. For example, the change of direction ability test (CODA) is proposed by FIFA to assess the ability of assistant referees' change of direction [7]. However, a trivial association between match performance and the time to complete the CODA test has been reported. Additionally, data quality about the methodological protocol used was not reported [70]. Furthermore, FIFA adopted the repeated sprint ability test (6 × 40 m) as an indicator of short-maximal outputs [7], but non-significant associations with mean heart rate during the match were found in 11 elite soccer referees [22]. FIFA also proposed a dynamic YOYO protocol that claims scientific validation. The assessment of physical capacity in soccer referees requires particular attention since studies using field protocols investigated match-related fatigue in soccer referees [43, 52, 53]. Evidence suggests that post-match 15-m and 30-m sprint performance [51] and isometric strength of non-dominant limb [53] were significantly lower compared with pre-match assessments; however, reductions were not observed in jump ability performance [52]. In a sample of 8 referees and 16 assistant referees, blood lactate increased by 1.7 mmol l^−1^ and 0.4 mmol l^−1^, respectively. Although the limited data indicated that referees finished the match with fatigue, muscle glycogen needs to be evaluated [130].

Fat mass had a negative impact on distance covered during the YOYO test and repeated sprint ability test [77]. Referees had lower values of fat mass percentage than assistant referees [62–64, 67], and younger referees tended to possess lower body fat than older referees [61–63]. The included studies tended to form a consensus that 15% fat mass is an acceptable value for referees. The Portuguese Soccer Federation provides bonuses to referees that achieve desirable fat mass percentage estimated from four skinfolds (biceps, triceps, subscapular and supra-iliac) [128]. However, possessing favourable body composition is not a prerequisite for international referees [7]. Additionally, the equation derived from 43 elite international soccer referees used two skinfolds (iliac crest and abdominal) and could be used to predict fat mass percentage in referees [66], but further research is needed before this predictive model can be adopted. It is also recommended that values are adjusted for referees and assistant referees given the difference in movement demands, and subsequent energy expenditure and physiological profiles likely influencing body composition.

Height was documented as an important characteristic to discriminate referees and assistant referees [60]. It was associated with authority and effectiveness during the match [59] as well as match performance [24]. However, data points of height and weight of the studies combined in the present review did not suggest a trend to distinguish between referees and assistant referees. Moreover, body size does not appear to discriminate referees of different competitive levels.

Energy and macronutrient intake in the three studies [72–74] suggested that energy intake ranged from 2409 kcal day^−1^ to 2819 kcal day^−1^. Additionally, for two of these studies, the Portuguese [72] and Spanish soccer referees [73] did not meet the recommendations for carbohydrate intake. Although the recommendations of soccer players have been generalized to referees [26], this point needs future consideration. In contrast with soccer players [131], no studies have determined the energy expenditure using the doubly labelled water method in referees, despite being considered the gold standard for measuring daily energy expenditure. Consequently, nutrition guidelines developed for soccer players should not be generalized to referees before detailed analyses are conducted.

Comparison of soccer referees and players also showed difference in maximal oxygen uptake [81, 85]. The referees attained lower values of maximal oxygen uptake but were typically older than the soccer players. Therefore, physiological parameters of aerobic fitness need to be frequently analysed among referees in order to ensure that they are able to reach aerobic levels comparable to soccer players [81]. Aerobic fitness also discriminated male and female soccer referees. Using an incremental running test, the maximal oxygen uptake in elite male referees was 3.98 l min^−1^, while elite female referees attained 2.94 l min^−1^ [83]. This study highlights the importance of aerobic training among female referees, especially if they are appointed for competitions in male soccer. This is even more important given that 6 female referees (3 referees and 3 assistant referees) were included on the list of 36 referees who participated in the World Cup hosted in Qatar 2022. It is also projected that there will be a higher number of female referees in male soccer moving forward. However, the literature in female referees is limited, and further research is required.

Although a considerable number of studies were extracted in the current review, a possible limitation is the inclusion of studies written solely in English. Additionally, studies investigating decision making, psychological aspects or visual parameters were not considered, but are key components for refereeing performance [132, 133]. The studies included in the present review also present limitations that should be acknowledged. Most of the studies were observational in design, which does not assess a cause–effect relationship. Furthermore, a negligible number of studies adequately determined the sample size, and the longitudinal studies ignored intra-individual variability.

## Conclusions

Older referees maintained the same distances from the ball, despite demonstrating reductions in running output. Therefore, age appears to be a poor criterion for classifying soccer referees. Match performance is affected by many factors and thus should not be exclusively used to validate field protocols. Height and weight were not useful in differentiating between soccer referees of different levels. The assessment of physical fitness warrants further attention given that there are high levels of fatigue that are present during the latter stages of matches. In order to attain ideal levels of body composition, future studies need to provide guidelines for daily energy expenditure and nutritional intake.

### Supplementary Information

**Additional file 1: Table S1.** Summary of study characteristics (sample, country, body size and composition) and purpose.

**Additional file 2: Table S2.** Studies that reported height, weight or fat mass.

**Additional file 3.** Risk of bias for each study using the tools proposed by the National Institutes of Health.

**Additional file 4: Table S4**. Tests often used among soccer referees.

## Data Availability

The authors confirm that the data supporting the findings of this study are available within the article and its additional files.

## Abbreviations

UEFA: Union of European Football Association
PRISMA: Preferred reporting items for systematic review
CODA: Change of direction ability
