# Supplementary material for: A Systematic Review of the Physical, Physiological, Nutritional and Anthropometric Profiles of Soccer Referees
Source: Sports Med Open. 2023 Aug 10;9:72. doi: 10.1186/s40798-023-00610-7 (PMC10415246; doi:10.1186/s40798-023-00610-7)
Supplement: Supplementary file 1 — Additional file 1: Table S1. Summary of study characteristics (sample, country, body size and composition) and purpose. [file 40798_2023_610_MOESM1_ESM.docx]

| Study | Sample | Country | Age (years) | Height (m or cm) | Weight (kg) | % fat mass | Purpose |
| --- | --- | --- | --- | --- | --- | --- | --- |
| Catterall et al. [109] | 14 | England |  |  |  |  | describe internal and external loads |
| Johnston and McNaughton [110] | 10 | Australia | 38.1±3.8 |  |  |  | describe internal and external loads |
| Castagna and D’Ottavio [33] | 8 | Italy | 37.6±3.4 | 182.9±4.5 | 77.6±6.8 |  | examine the relationship between aerobic power and match intensity |
| D’Ottavio and Castagna, [111] | 33 | Italy | 37.8±2.1 |  |  |  | describe internal load |
| D’Ottavio and Castagna [11] | 18 | Italy | 37.5±2.4 |  |  |  | describe internal and external loads |
| Krustrup and Bangsbo [34] | 12 | Denmark | 29-47 | 1.69-1.95 | 69.3-101.6 |  | describe internal and external loads,  validity of intermittent test, effect of intermittent training on match performance |
| Castagna et al. [35] | 8 | Italy | 37.6±3.4 | 182.9±4.5 | 77.6±7.0 |  | examine relationship between match activity and blood lactate thresholds |
| Castagna et al. [12] | 22 | Italy | 37.0±2.4 | 182.0±4.5 | 76.8±7.0 |  | examine relationship between match activity and field protocols |
| Krustrup et al. [13] | 15 AR | Denmark | 32-47 | 1.67-1.91 | 69.1-98.4 |  | describe internal and external load, relationship between physical capacity and match analysis. |
| Castagna and Abt [98] | 14 | Italy | 37.0±2.4 | 182.0±5.5 | 77.9±7.0 |  | variation in high intensity activities across the games |
| Castagna et al. [101] | 14 international | Italy | 38.3±3.0 | 182.0±6.5 | 78.8±7.0 |  | describe the internal load of international referees consider different competitions |
|  | 14 national |  | 37.0±3.0 | 182.0±3.5 | 77.1±6.5 |  |  |
| Helsen and Bultynck [112] | 17 |  | 40.2±3.9 | 1.82±0.06 | 79.7±9.2 |  | examine external load and perceptual-cognitive demands |
|  | 17 AR |  | 41.3±2.8 | 1.77±0.08 | 75.3±8.9 |  |  |
| Weston et al. [54] | 7 international | Belgium | 37.8±4.1 |  |  |  | examine the effects of high intensity training on heart rate |
|  | 7 national |  | 39.3±4.2 |  |  |  |  |
|  |  |  |  |  |  |  |  |

**Supplementary Table S1.** Summary of study characteristics (sample, country, body size and composition) and purpose.

| Study | Sample | Country | Age (years) | Height (m or cm) | Weight (kg) | % fat mass | Purpose |
| --- | --- | --- | --- | --- | --- | --- | --- |
| Castagna et al. [45] | 14 top-level | Italy | 37.5±4.5 | 184.3±5.2 | 78.5±5.6 |  | differences between competitive levels on the field tests |
|  | 14 medium-level |  | 27.8±3.2 | 182.3±4.2 | 78.1±2.3 |  |  |
|  | 14 low-level |  | 24.8±1.2 | 184.5±2.2 | 77.6±3.6 |  |  |
| Castagna et al. [15] | 12 young | Italy | 33.5±2.5 | 181.0±2.5 | 77.5±2.1 |  | age variation in field and laboratory tests |
|  | 14 average |  | 37.5±1.0 | 182.0±1.0 | 76.8±1.8 |  |  |
|  | 10 old |  | 42.0±1.0 | 180.0±2.5 | 77.0±2.1 |  |  |
| Weston et al. [113] | 18 | England | 41.8 |  |  |  | examine the relationship between heart rate and ratings of perceived exertion, impact of match activity and experience and workloads |
| Casajus and Castagna [42] | 15 young | Spain | 30.4±1.5 | 178.7±5.4 | 73.4±6.1 | SK:11.1±0.6 | investigate age variation in field and laboratory tests |
|  | 17 average |  | 35.8±1.2 | 179.4±4.8 | 77.3±6.9 | SK:11.1±0.5 |  |
|  | 13 old |  | 40.4±2.5 | 176.4±4.5 | 74.3±6.2 | SK:11.9±0.6 |  |
|  | all sample |  | 35.5±4.4 | 178.3±5.0 | 75.1±6.6 | SK:11.3±2.2 |  |
| Tessitore et al. [14] | 10 | Italy | 23.8±1.8 | 182.0±6.6 | 74.7±7.4 |  | examine changes in jump performance during the match |
| Weston et al. [5] | 19 | England | 40.1±4.9 |  | 84.1±8.9 |  | examine internal load regarding competitive season and match-to-match variation |
| da Silva et al. [68] |  | Brazil | 38.9±3.8 | 1.80±0.07 | 86.1±7.1 |  | examine energy expenditure |
| Galanti et al. [78] | 120 | Italy | 32.0±3.3 | 184.0±5.7 | 77.2±6.6 |  | describe cardiac outputs |
| Mallo et al. [48] | 22 AR |  | 34.0±3.3 | 175.8±7.3 | 73.3±8.0 |  | describe internal load and relationship with field protocols |
| Bizzini et al.* | 81 F |  | 35.0±4.4 | 1.66±0.06 | 59.0±6.0 |  | investigate injuries and musculoskeletal problems |
| Bizzini et al. [88] | 66 | Swiss | 36.0±5.3 | 180.0±6.0 | 76.0±6.0 |  | investigate injuries and musculoskeletal problems |
| Krustrup et al. [89] | 15 |  | 42 | 188 | 82.5 |  | examine internal and external load |
|  | 15 AR |  | 43 | 181 | 77.8 |  |  |
| Mallo et al. [22] | 11 |  | 39.3±3.4 | 1.83±0.04 | 78.8±4.5 |  | describe internal load, examine the relationship between heart rate and internal load, test the association between field protocols and match activities |
|  |  |  |  |  |  |  |  |

| Study | Sample | Country | Age (years) | Height (m or cm) | Weight (kg) | % fat mass | Purpose |
| --- | --- | --- | --- | --- | --- | --- | --- |
| Mallo et al. [23] | 18 AR |  | 38.7±4.8 | 1.77±0.07 | 75.1±8.6 |  | examine the relationship between internal load and heart rate, examine the effect of the competition level and ball position on internal load |
| Weston et al. [37] | 17 | England | 40.0±5.1 |  | 82.8±10.0 |  | test the association of FIFA tests on internal load |
| Catteuw et al. [114] | 48 AR | England | 41.8±5.7 |  |  |  | examine factors that can influence offside decision |
| Weston et al. [20] | 22 | England |  |  |  |  | examine the effects of age on match demands |
| Mallo et al. [107] | 14 F AR |  | 34.8±3.5 | 1.64±0.05 | 58.1±7.4 |  | describe internal load |
| Mallo et al. [108] | 10 F |  | 35.3±4.3 | 168.4±3.9 | 62.5±6.4 |  | describe internal load |
| Caballero et al. [38] | 54 | Spain | 28.5±6.4 | 1.76±0.07 | 77.3±10.7 |  | describe echocardiography parameters |
| Caballero et al. [17] | 22 | Spain | 26.2±5.0 | 1.77±0.07 | 77.1±12.6 |  | describe a physiological profile |
| Castagna et al. [46] | 100 AR | Italy: Serie A-B | 37.0±2.9 | 1.78±6.9 | 74.0±6.9 |  | applicability of field tests |
|  |  | Italy: Legal Pro | 34.0±2.0 | 1.77±4.9 | 75.0±4.9 |  |  |
| Di Salvo et al. [115] | 68 | England and international competitions |  |  |  |  | comparing internal load in different competitions |
|  | 170 AR |  |  |  |  |  |  |
| Silva et al. [69] | 215 | Brasil | 33.7±5.7 | 177.5±6.1 | 78.5±10.3 |  | examine age-variation in body composition |
| Silva et al. [61] | 10 | Brasil | 37.0±2.9 | 1.80±0.02 | 85.0±2.1 |  | influence of hydration on performance and examine the impact of fluid ingested |
| Weston et al. [100] | 12 | England | 39.5±6.2 |  | 81.2±8.6 |  | examine differences in work-rate loads between players and referees |
| Weston et al. [106] | 59 | England |  |  |  |  | examine short and long-term match variability on internal load |
| Weston et al. [116] | 18 | England | 40.0±5.0 |  | 82.8±10.0 |  | describe variation in internal load within-match and across the season |
|  |  |  |  |  |  |  |  |

| Study | Sample | Country | Age (years) | Height (m or cm) | Weight (kg) | % fat mass | Purpose |
| --- | --- | --- | --- | --- | --- | --- | --- |
| Barbero-Álvarez et al. [97] | 7 |  | 40.0±2.2 | 1.75±0.42 | 68.0±6.8 |  | examine the internal and external loads |
|  | 7 AR |  | 36.0±5.2 | 1.74±0.35 | 76.1±6.3 |  |  |
| Bizzini et al. [90] | 90 |  | 39.1±3.9 | 1.78±0.07 | 77.4±7.8 |  | describe precompetition medical assessment |
|  | 30 |  |  |  |  |  |  |
|  | 60 AR |  |  |  |  |  |  |
| Boullosa et al. [50] | 11 | Spain | 26.0±5.0 | 179.0±3.0 | 75.1±3.8 |  | examine the impact of a soccer match on the cardiac autonomic control |
|  | 5 F |  | 22.0±3.0 | 158.0±9.0 | 59.3±4.8 |  |  |
| Castagna et al. [8] | 245 AR | Italy and Deutch |  |  |  |  | test data quality (validity and reliability) of the assistant referee endurance test |
| Mallo et al. [117] | 10 | Denmark | 39.4±2.4 | 182.5±5.9 | 78.4±4.6 |  | examine the effect of positioning on the decision |
|  | 20 AR |  | 37.3±4.0 | 178.2±7.1 | 74.7±7.9 |  |  |
| Stulp et al. [59] | 38 |  | 41.4±5.8 | 179.7±5.5 |  |  | association between height and authority |
| Costa et al. [118] | 11 | Brazil | 36.2±7.5 |  |  | NR:14.6±5.6 | Describe the internal and external loads |
|  | 22 AR |  | 34.0±8.0 | 181.0±17.0 | 81.0±18.0 |  |  |
| Barbero-Álvarez et al. [119] | 7 |  | 40.0±2.2 | 1.75±0.02 | 68.0±6.8 |  | define repeated sprint sequences and repeated sprint ability |
|  | 7 AR |  | 36.0±5.2 | 1.74±0.03 | 76.1±6.3 |  |  |
| Casajus et al. [62] | 144 young | Spain | 28.8±2.9 | 179.8±5.9 | 71.9±5.9 | BIA:7.4±2.4 | examine body composition according to age group and competitive level |
|  | 66 average |  | 36.0±1.7 | 178.3±5.8 | 73.0±6.7 | BIA:8.4±2.7 |  |
|  | 33 senior |  | 41.2±1.7 | 177.5±5.9 | 72.7±6.1 | BIA:8.8±2.7 |  |
|  | all sample |  | 32.4±5.3 | 179.1±5.9 | 72.3±6.2 | BIA:7.8±2.6 |  |
|  |  |  |  |  |  |  |  |

| Study | Sample | Country | Age (years) | Height (m or cm) | Weight (kg) | % fat mass | Purpose |
| --- | --- | --- | --- | --- | --- | --- | --- |
| Palmer et al. [86] | 7 | U.S. | 36.0±2.4 | 178.5±3.0 | 82.1±3.6 |  | examine the influence of strength to discriminate referees |
|  | 9 |  | 34.1±2.1 | 181.2±1.8 | 84.3±2.5 |  |  |
| Pietraszewski et al. [91] | 30 |  | 32.7±3.7 | 182.6±4.6 | 80.0±15.6 |  | examine executive attention |
|  | 23 AR |  | 34.9±3.5 | 181.8±5.7 | 81.1±7.7 |  |  |
| Silva et al. [81] | 10 | Brazil | 24.1±3.4 | 178.2±7.4 | 78.0±8.3 | SK:10.8±2.6 | comparisons between maximal oxygen uptake and anthropometric characteristics among players and referees |
| Teixeira et al. [72] | 83 | Portugal | 34.4±5.6 | 1.78±0.06 | 77.8±6.7 |  | describe the dietary intake |
| Martínez Reñon and Collado [73] | 35 | Spain | 24.7±9.9 | 178±6.5 | 73.1±6.5 |  | describe the dietary intake |
| Metz et al. [74] | 6 |  | 22.8±6.8 |  |  |  | examine the impact of soccer game on energy intake and appetite feedings |
| Casajús et al. [63] | 130 young | Spain | 28.8±2.7 | 179.6±6.1 | 72.1±5.9 | BIA:11.0±2.8 | assess fat mass variation across the season |
|  | 71 middle |  | 35.9±1.7 | 177.8±6.2 | 73.0±6.9 | BIA:11.9±2.9 |  |
|  | 27 senior |  | 41.5±1.9 | 178.6±5,9 | 74.9±6.7 | BIA:12.8±3.1 |  |
|  | all sample |  | 32.5±5.1 | 178.9±6.1 | 72.7±6.3 | BIA:11.5±2.9 |  |
| Castillo et al. [38] | 20 | Spain | 28.4±7.3 | 177.8±6.7 | 74.3±8.3 |  | analyse the impact of the match on field protocols |
| Castillo et al. [51] | 8 | Spain | 25.6±5.3 | 182.8±6.6 | 77.0±8.9 |  | examine the influence of a soccer match in performance and physiological parameters |
|  | 8 AR |  | 32.3±9.6 | 175.4±4.1 | 74.4±8.3 |  |  |
| Castillo et al. [43] | 23 | Spain | 30.0±6.7 | 176.8±6.1 | 73.3±8.1 |  | describe field and laboratory assessments, examine variation between competitive level, relate sprint, change of direction and endurance |
|  | 22 AR |  | 29.2±8.9 | 175.0±5.6 | 73.3±7.3 |  |  |
|  |  |  |  |  |  |  |  |

| Study | Sample | Country | Age (years) | Height (m or cm) | Weight (kg) | % fat mass | Purpose |
| --- | --- | --- | --- | --- | --- | --- | --- |
| de Oliveira et al. [92] | 17 | Brazil | 35.0±4.7 | 1.82±0.07 | 83.0±7.5 |  | describe the prevalence of injuries |
|  | 19 |  | 31.0±5.9 | 1.82±0.06 | 86.0±7.1 |  |  |
| Gomez-Carmona and Pino-Ortega [120] | 6 | Spain | 21.2±1.0 | 175.7±4.2 | 65.7±4.7 |  | describe internal and external load, examine the influence on decision making |
| Mazaheri et al. [82] | 77 | Iran | 40.0±3.8 | 178.0±5.8 | 74.9±8.1 | BIA:20.7±3.9 | association between cardiorespiratory parameters, body composition with performance |
| Paes and Fernandez [70] | 10 | Brazil | 29.0±7.8 | 1.78±0.07 | 77.5±6.2 | NR:19.9±2.1 | measure energy expenditure in specific movements under different conditions |
| Yanci et al. [126] | 41 | Spain |  |  |  |  | examine the relationship between change-of-direction and acceleration, compare these parameters by competitive level |
|  | national league |  | 28.4±1.4 | 177.4±0.04 | 73.5±7.8 |  |  |
|  | provincial league |  | 29.5±1.9 | 176.8±0.04 | 77.7±10.8 |  |  |
| Bozdogan et al. [77] | 158 | Turkey | 31.8±4.2 |  | 77.0±6.5 | BIA:10.6±3.0 | examine the impact of body size and composition on field protocols and heart rate |
|  | 55 AR |  | 37.4±3.3 |  | 77.7±10.8 | BIA:11.6±3.3 |  |
| Castagna et al. [128] | 51 |  | 38.4±3.3 | 181.0±5.6 | 76.8±6.8 | NR:20.4±3.6 | examine the timing effect of RPE recall |
| Castillo et al. [18] | 23 | Spain | 30.0±6.7 | 176.8±6.1 | 73.3±8.1 |  | examine the effects of transitory period on field performance |
| Castillo et al. [121] | 20 | Spain | 27.7±6.2 | 177.6±6.7 | 74.1±8.5 |  | describe internal and external load, examine the agreement between internal and external load |
|  | 43 AR |  | 30.7±9.6 | 176.2±5.6 | 75.1±7.8 |  |  |
| Dolanski et al. [122] | 10 | Poland | 26-30 | 177-183 | 68-78 |  | examine internal load |
|  | 10 AR |  | 25-28 | 183-190 | 76-83 |  |  |
| Fernández-Elías et al. [123] | 14 | Spain | 25.8±3.8 | 1.79±0.03 | 74.4±2.3 |  | examine internal and external loads |
| Castagna et al. [83] | 51 |  | 38.4±3.3 | 181.0±5.6 | 76.0±6.8 | NR:20.4±3.6 | sex differences in aerobic fitness |
|  | 40 F |  | 34.2±3.5 | 169.0±5.3 | 61.0±6.0 | NR:24.9±4.0 |  |
| Castillo et al. [102] | 20 | Spain | 27.7±6.2 | 177.6±6.7 | 74.1±8.3 |  | examine internal and external loads in different competitive games |
|  |  |  |  |  |  |  |  |

| Study | Sample | Country | Age (years) | Height (m or cm) | Weight (kg) | % fat mass | Purpose |
| --- | --- | --- | --- | --- | --- | --- | --- |
| Castillo et al. [52] | 18 | Spain | 27.2±5.7 | 177.8±6.7 | 74.1±8.1 |  | investigate the effect of soccer matches on jumping performance |
| Gianturco et al. [47] | 40 | Italy | 20.5±1.5 | 1.78±0.1 | 74.2±5.6 |  | examine relationship between foot characteristics and YOYO test |
| Coffi et al. [80] | 37 | Benin | 29.6±6.8 | 173.0±1.1 | 67.7±1.3 |  | describe echocardiograph parameters |
| Riiser et al. [39] | 9 | Norway | 38.0±7.0 | 182.0±6.0 | 76.0±8.0 |  | association between field protocols and match load |
|  | 21 AR |  | 37.0±8.0 | 182.0±8.0 | 77.0±7.0 |  |  |
| Sánchez-Garcia et al. [48] | 23 | Spain | 24.0±5.0 | 179.0±5.0 | 74.7±9.8 |  | examine the association between field protocols |
| Talovic et al. [77] | 60 | Bosnia and Herzegovina |  |  |  |  | assess strength asymmetry in different levels of competition |
|  | Premier League |  | 32.2±4.0 | 183.9±4.9 | 80.5±6.9 | BIA:10.9±2.2 |  |
|  | First League |  | 34.9±3.2 | 184.6±8.1 | 82.7±9.9 | BIA:9.7±3.0 |  |
| Yanaoka et al. [58] | 10 | Japan | 22.0±1.0 | 173.6±5.8 | 67.2±6.4 |  | examine the influence of re-warm up on performance |
| Banda et al. [60] | 41 | Zimbabwe | 34.9±5.1 | 175.7±6.9 | 70.5±10.5 | SK:12.0±2.6 | describe and compare body composition |
|  | 21 |  | 35.0±5.6 | 177.8±7.3 | 71.8±11.6 | SK:12.2±2.6 |  |
|  | 20 AR |  | 34.8±4.9 | 173.5±5.7 | 69.2±9.3 | SK:11.7±2.6 |  |
| Casajus et al. [64] | 30 | Spain | 33.2±5.0 | 180.6±4.9 | 74.4±5.7 | DXA:15.6±2.0 | test the agreement between methods to assess body composition |
|  |  |  |  |  |  | BIA:11.7±2.6 |  |
|  | 36 AR |  |  | 177.3±6.4 | 74.5±7.5 | DXA:17.8±2.8 |  |
|  |  |  |  |  |  | BIA:13.9±2.8 |  |
| Castagna et al. [84] | 52 elite | Italy | 38.4±3.3 | 181.0±5.6 | 76.8±6.8 | NR:20.4±3.6 | examine aerobic outputs |
|  | 104 AR |  | 37.8±4.1 | 176.9±7.5 | 72.1±7.4 | NR:19.2±3.6 |  |
| Castillo et al. [40] | 25 | Spain | 28.0±7.0 | 178.0±7.0 | 73.2±8.6 |  | compare internal and external load, with field assessments |
|  | 19 AR |  | 27.0±8.0 | 177.0±6.0 | 74.2±6.6 |  |  |
| Castillo et al. [65] | 14 | Spain | 28.8±5.1 | 179.0±7.0 | 73.1±6.5 | SK:25.0±3.0 | examine the effect of 10-week competitive period on body composition |
|  |  |  |  |  |  |  |  |

| Study | Sample | Country | Age (years) | Height (m or cm) | Weight (kg) | % fat mass | Purpose |
| --- | --- | --- | --- | --- | --- | --- | --- |
| Joo and Jee [123] | 14 | Korea | 37.5±4.3 |  |  |  | compare the activity of players and referees |
| Malaguti et al. [71] | 71 |  | 33.7±5.3 |  |  |  | evaluate nutritional knowledge on supplements |
| Maslennikov et al. [55] | 50 | Russia |  |  |  |  | describe internal and external load |
| Santos-Silva et al. [85] | 50 | Brazil | 34.8±4.6 | 179.0±6.0 | 75.9±8.4 |  | comparison between soccer and referees on aerobic performance |
| Schmidt et al. [93] | 33 | Brazil | 29.2±3.8 | 1.79±0.73 | 76.8±4.8 |  | examine attentional performance after FIFA-Test |
|  | 10 AR |  | 24.5±2.4 | 1.74±0.60 | 70.6±4.3 |  |  |
|  | 10 F AR |  | 29.3±4.0 | 1.66±0.55 | 60.5±3.9 |  |  |
| Gacek et al. [75] | 138 | Poland | 31.7±8.9 |  |  |  | analyse the frequencies of food intake |
| Mascherini et al. [76] | 60 | Italy | 39.2±4.2 | 1.82±0.6 | 77.1±6.8 | SK:11.4±2.5 | describe body composition and nutritional intake |
|  |  |  |  |  |  | BIA:17.1±1.8 |  |
| McCarrick et al. [24] | 61 | England | 37.4±7.9 | 176.9±9.8 |  |  | test the relationship between height and authority |
| Meckel et al. [44] | 17 first division |  | 36.0±5.6 | 181.4±4.2 | 75.1±6.5 |  | examine RSA, age-variation and competitive level |
|  | 23 first division AR |  | 36.3±5.1 | 179.4±6.7 | 75.8±9.0 |  |  |
|  | 10 second division |  | 26.8±2.2 | 182.3±5.2 | 76.5±3.3 |  |  |
|  | 10 second division AR |  | 31.3±3.4 | 176.6±7.6 | 72.2±7.0 |  |  |
| Muniroglu and Suback [56] | 94 | Turkey | 24.3±2.9 | 180.4±6.1 | 72.5±7.3 |  | test the effects of carrying a flag on sprint performance |
| Muscella et al. [16] | 17 |  | 20.5±5.6 | 176.2±4.2 | 66.5±3.1 | SK:13.1±0.7 | examine variation across the season in physical performance |
|  | 16 |  | 23.4±1.7 | 180.3±2.4 | 70.2±4.8 | SK:13.8±1.2 |  |
|  | 16 |  | 35.5±4.2 | 179.7±3.3 | 77.2±5.2 | SK:14.2±3.4 |  |
|  |  |  |  |  |  |  |  |

| Study | Sample | Country | Age (years) | Height (m or cm) | Weight (kg) | % fat mass | Purpose |
| --- | --- | --- | --- | --- | --- | --- | --- |
| Petri et al. [66] | 43 | Italy | 38.8±3.6 | 1.80±0.1 | 75.7±6.5 | DXA:18.2±4.1 | describe body composition and test the agreement between methods |
| Senecal et al. [94] | 8 | Canada | 30.4±5.4 | 1.77±0.05 | 76.6±7.1 |  | examine the effect of exercise on attention |
|  | 4 F |  | 26.3±2.3 | 1.64±0.08 | 62.4±4.1 |  |  |
| Aguilar et al. [95] | 256 | Spain | 23.7±3.4 | 177.5±8.9 | 72.7±8.9 |  | examine the association between self-efficacy, group and experience |
| Baydemir et al. [57] | 25 | Turkey | 23.9±2.1 | 181.2±2.0 | 74.4±2.5 |  | examine the effects of high-intensity interval training on FIFA athletic test |
| Fernández-Ruiz et al. [53] | 12 | Spain | 25.3±3.3 | 1.81±0.05 | 75.5±12.9 |  | effects of soccer match on strength outputs |
|  | 23 AR |  | 25.0±9.9 | 1.78±0.02 | 73.0±9.9 |  |  |
| Lopez-Garcia et al. [67] | 9 | Mexico | 24.3±3.5 | 175.5±5.6 | 71.3±8.6 |  | describe body size and composition |
|  | 13 AR |  | 25.7±3.5 | 172.6±6.1 | 74.1±7.2 |  |  |
| Muscella et al. [96] | 29 |  | 22.5±3.1 | 176.5±4.2 | 70.5±3.1 | SK:13.6±0.7 | examine the effects of soccer season on hormonal parameters and field protocols |
| Oazeta et al. [124] | 23 | Spain | 25.7±3.3 | 173.4±3.8 | 64.9±5.8 |  | examine internal and external load |
|  | 46 AR |  | 23.1±4.2 | 178.2±4.3 | 72.8±6.7 |  |  |
| Romano et al. [127] | 30 | Italy | 22.2±1.8 | 180.4±6.3 | 73.7±7.4 |  | examine association between internal, external loads and match performance |
| Castillo-Rodriguez et al. [103] | 153 | Spain | 23.2±4.8 |  |  |  | examine relationship between internal load, external load and psychological characteristics |
| Moreno-Perez et al., 2021 [105] | 17 | Spain | 38.7±3.5 | 181.9±4.1 | 74.7±3.6 |  | examine the effects of match congestion on load (internal and external). |
| Castagna et al. [49] | 38 | Asia | 28.5±1.5 | 178.0±5.1 | 69.0±7.3 | NR:17.2±2.9 | describe normative data about fitness performance |
| Martínez-Torremocha et al. [104] | 19 first division | Spain | 37.8±4.4 | 182.7±5.7 | 75.1±4.5 |  | compare internal and external match load in two different competitive levels |
|  | 21 second division |  | 34.4±3.6 | 182.6±4.1 | 75.2±4.5 |  |  |
| Preissler et al. [41] | 14 | Brazil | 35.7±3.9 | 182.6±5.6 | 85.8±6.9 |  | compare internal and external load on physical tests and matches |
|  |  |  |  |  |  |  |  |

F (female); AR (assistant referees); SK (skinfolds); BIA (biompedance); DXA (dual-energy X-ray absorptiometry); NR (not reported). *Bizzini M, Junge A, Bahr R, Dvorak J. Female soccer referees selected for the FIFA Women's World Cup 2007: survey of injuries and musculoskeletal problems. Br J Sports Med. 2009;43(12):936-942.
