# Supplementary material for: A Systematic Review of the Physical, Physiological, Nutritional and Anthropometric Profiles of Soccer Referees
Source: Sports Med Open. 2023 Aug 10;9:72. doi: 10.1186/s40798-023-00610-7 (PMC10415246; doi:10.1186/s40798-023-00610-7)
Supplement: Supplementary file 2 — Additional file 2: Table S2. Studies that reported height, weight or fat mass. [file 40798_2023_610_MOESM2_ESM.docx]

**Supplementary Table S2.** Studies that reported height, weight or fat mass.

| Study | Results/main findings | Practical applications |
| --- | --- | --- |
| Bizzini et al.* | Half of the sample reported at least one injury, which impacted time loss during the game. Musculoskeletal problems were reported in 79% of the participants. During the competition (World Cup), 39% of the sample suffered injuries, and 33% had musculoskeletal problems. Most injuries and problems occurred in lower limbs (hamstrings, quadriceps, calf and ankle). | Female-specific programs to prevent injuries should be developed. |
| Bizzini et al. [88] | A retrospective questionnaire showed that injuries tend to occur during training sessions. Considering a 12-month period, referees reported injuries and musculoskeletal complaints related to refereeing – injuries and musculoskeletal complaints were frequent in lower limbs. Negligible differences were noted between referees and assistant referees. | The current study suggested that prevention programs for referees should be created. |
| Bizzini et al. [90] | Cardiac pathologies were frequent among referees. However, the follow-up analysis concluded that the examination was within the expected values. A potential factor in explaining pathology could be age. | The high risk of occult ischaemic cardiac suggested that an electrocardiogram should be performed on soccer referees. |
| Pietraszewski et al. [91] | Assistant referees showed the best precision index than referees. Referees tended to make more errors on the test. The precision was also influenced by competitive level and age. | Sports scientists, soccer governing bodies and those responsible for developmental programs should know that executive functions differ in referees and assistant referees. |
| de Oliveira et al. [92] | Injuries among Brazilian referees were mainly classified as strain, sprain and fracture and tended to occur in training and physical tests. | Most referees train without supervision; therefore, specialized professionals should be responsible for the training program designs. |
| Schmidt et al. [93] | At baseline, 17 referees (32% of the total sample) showed attention problems. Analysing only the participants who passed on the first Continuous Visual Attention Test and FIFA battery protocols, 44% of participants showed a performance decrement on the second assessment of the Continuous Visual Attention Test, particularly in velocity reaction time. | Referees may were not prepared for the attentional demands of soccer matches. Consequently, FIFA battery protocols should include executive attentional tasks. |
| Senecal et al. [94] | Performance, expressed as the time to complete the Stroop Test, improved significantly at moderate (d = 0.77) and maximal (d = 1.09) intensities compared to the pre-test session. | The present results showed that referees focused on goal-oriented processing during maximal intensities. |
| Aguilar et al. [95] | Generally, age, experience and competitive level were related to self-efficacy among referees. Self-efficacy considered four domains: game knowledge, decision-making, pressure and communication. | The results were useful in understanding and prevent burnout in lower-category and may need intervention programs related to psychological skills. |
|  |  |  |

*Bizzini M, Junge A, Bahr R, Dvorak J. Female soccer referees selected for the FIFA Women's World Cup 2007: survey of injuries and musculoskeletal problems. Br J Sports Med. 2009;43(12):936-942.
