# Supplementary material for: A Systematic Review of the Physical, Physiological, Nutritional and Anthropometric Profiles of Soccer Referees
Source: Sports Med Open. 2023 Aug 10;9:72. doi: 10.1186/s40798-023-00610-7 (PMC10415246; doi:10.1186/s40798-023-00610-7)
Supplement: Supplementary file 3 — Additional file 3. Risk of bias for each study using the tools proposed by the National Institutes of Health. [file 40798_2023_610_MOESM3_ESM.docx]

**Supplementary Table S3.** Questions from the Quality Assessment Tool for Observational Cohort and Cross-Sectional Studies checklist used to evaluate methodological quality of the included articles.

| Criteria no. | Question |
| --- | --- |
| 1 | Was the research question or objective in this paper clearly stated? |
| 2 | Was the study population clearly specified and defined? |
| 3 | Was the participation rate of eligible persons at least 50%? |
| 4 | Were all the subjects selected or recruited from the same or similar populations (including the same time period)? Were inclusion and exclusion criteria for being in the study prespecified and applied uniformly to all participants? |
| 5 | Was a sample size justification, power description, or variance and effect estimates provided? |
| 6 | For the analyses in this paper, were the exposure(s) of interest measured prior to the outcome(s) being measured? |
| 7 | Was the timeframe sufficient so that one could reasonably expect to see an association between exposure and outcome if it existed? |
| 8 | For exposures that can vary in amount or level, did the study examine different levels of the exposure as related to the outcome (e.g., categories of exposure, or exposure measured as continuous variable)? |
| 9 | Were the exposure measures (independent variables) clearly defined, valid, reliable, and implemented consistently across all study participants? |
| 10 | Was the exposure(s) assessed more than once over time? |
| 11 | Were the outcome measures (dependent variables) clearly defined, valid, reliable, and implemented consistently across all study participants? |
| 12 | Were the outcome assessors blinded to the exposure status of participants? |
| 13 | Was loss to follow-up after baseline 20% or less? |
| 14 | Were key potential confounding variables measured and adjusted statistically for their impact on the relationship between exposure(s) and outcome(s)? |
|  | **Quality Rating (Good, Fair, or Poor)** |

**Legend:**

1-YES 2 - NO; 3 - cannot determine, not applicable or not reported

**Supplementary Table 3.** Results of methodological quality assessment for Observational Cohort and Cross-Sectional Studies.

| Study | Quality Assessment Tool for Observational Cohort and Cross-Sectional Studies checklist question number | | | | | | | | | | | | | | |
| --- | --- | --- | --- | --- | --- | --- | --- | --- | --- | --- | --- | --- | --- | --- | --- |
|  | 1 | 2 | 3 | 4 | 5 | 6 | 7 | 8 | 9 | 10 | 11 | 12 | 13 | 14 | Quality rating |
| Catterall et al. [109] | 1 | 1 | 1 | 1 | 2 | 3 | 3 | 3 | 1 | 2 | 1 | 3 | 3 | 1 | **F** |
| Johnston et al. [110] | 1 | 1 | 1 | 1 | 2 | 3 | 3 | 3 | 1 | 2 | 1 | 3 | 3 | 1 | **F** |
| Castagna et al. [33] | 1 | 1 | 1 | 1 | 2 | 3 | 3 | 3 | 1 | 1 | 1 | 3 | 2 | 1 | **G** |
| D’Ottavio et al. [111] | 1 | 1 | 1 | 1 | 2 | 3 | 1 | 3 | 1 | 1 | 1 | 3 | 2 | 1 | **G** |
| D’Ottavio et al. [11] | 1 | 1 | 1 | 1 | 2 | 3 | 3 | 3 | 1 | 3 | 1 | 3 | 3 | 1 | **F** |
| Krustrup et al. [34] | 1 | 1 | 1 | 2 | 2 | 3 | 3 | 3 | 1 | 3 | 1 | 3 | 3 | 1 | **G** |
| Castagna et al. [35] | 1 | 1 | 1 | 1 | 2 | 3 | 3 | 3 | 1 | 1 | 1 | 3 | 2 | 1 | **G** |
| Castagna et al. [12] | 1 | 1 | 1 | 1 | 2 | 3 | 3 | 3 | 1 | 1 | 1 | 3 | 2 | 1 | **G** |
| Krustrup et al. [13] | 1 | 1 | 1 | 1 | 2 | 3 | 3 | 3 | 1 | 1 | 1 | 3 | 2 | 1 | **G** |
| Castagna et al. [98] | 1 | 1 | 1 | 1 | 2 | 3 | 3 | 3 | 1 | 3 | 1 | 3 | 3 | 1 | **G** |
| Castagna et al. [101] | 1 | 1 | 1 | 2 | 2 | 3 | 3 | 3 | 1 | 3 | 1 | 3 | 3 | 1 | **G** |
| Helsen et al. [112] | 1 | 1 | 1 | 1 | 2 | 3 | 3 | 3 | 1 | 2 | 1 | 3 | 3 | 1 | **F** |
| Weston et al. [54] | 1 | 1 | 1 | 2 | 2 | 3 | 3 | 3 | 1 | 1 | 1 | 2 | 2 | 1 | **G** |
| Castagna et al. [45] | 1 | 1 | 1 | 2 | 2 | 3 | 3 | 3 | 1 | 2 | 1 | 3 | 3 | 1 | **F** |
| Weston et al. [113] | 1 | 1 | 1 | 1 | 2 | 3 | 3 | 3 | 1 | 2 | 1 | 3 | 3 | 1 | **G** |
| Casajus et al. [42] | 1 | 1 | 1 | 1 | 1 | 3 | 3 | 3 | 1 | 2 | 1 | 3 | 3 | 1 | **G** |
| Tessitore et al. [14] | 1 | 1 | 1 | 1 | 2 | 3 | 3 | 3 | 1 | 2 | 1 | 3 | 3 | 1 | **G** |
| Weston et al. [37] | 1 | 1 | 1 | 1 | 2 | 3 | 3 | 3 | 1 | 2 | 1 | 3 | 3 | 1 | **G** |
| da Silva et al. [67] | 1 | 2 | 1 | 1 | 2 | 3 | 3 | 3 | 1 | 2 | 1 | 3 | 3 | 1 | **F** |

| Study | Quality Assessment Tool for Observational Cohort and Cross-Sectional Studies checklist question number | | | | | | | | | | | | | | |
| --- | --- | --- | --- | --- | --- | --- | --- | --- | --- | --- | --- | --- | --- | --- | --- |
|  | 1 | 2 | 3 | 4 | 5 | 6 | 7 | 8 | 9 | 10 | 11 | 12 | 13 | 14 | Quality rating |
| Galanti et al. [78] | 2 | 1 | 1 | 1 | 2 | 3 | 3 | 3 | 1 | 2 | 1 | 3 | 3 | 2 | **P** |
| Mallo et al. [36] | 1 | 1 | 1 | 1 | 2 | 3 | 3 | 3 | 1 | 2 | 1 | 3 | 3 | 1 | **G** |
| Bizzini et al.* | 1 | 1 | 1 | 1 | 2 | 3 | 3 | 3 | 1 | 2 | 1 | 3 | 3 | 1 | **G** |
| Bizzini et al. [88] | 1 | 1 | 1 | 1 | 2 | 3 | 3 | 3 | 1 | 2 | 1 | 3 | 3 | 1 | **G** |
| Krustrup et al. [89] | 1 | 1 | 1 | 1 | 2 | 3 | 3 | 3 | 1 | 2 | 1 | 3 | 3 | 1 | **G** |
| Mallo et al. [22] | 1 | 1 | 1 | 1 | 2 | 3 | 3 | 3 | 1 | 2 | 1 | 3 | 3 | 1 | **G** |
| Mallo et al. [23] | 1 | 1 | 1 | 1 | 2 | 3 | 3 | 3 | 1 | 2 | 1 | 3 | 3 | 1 | **G** |
| Weston et al. [37] | 1 | 1 | 1 | 1 | 2 | 3 | 3 | 3 | 1 | 2 | 1 | 3 | 3 | 1 | **G** |
| Catteeuw et al. [114] | 1 | 1 | 1 | 1 | 2 | 3 | 3 | 3 | 1 | 2 | 1 | 3 | 3 | 1 | **G** |
| Mallo et al. [107] | 1 | 1 | 1 | 1 | 2 | 3 | 3 | 3 | 1 | 2 | 1 | 3 | 3 | 1 | **G** |
| Mallo et al. [108] | 1 | 1 | 1 | 1 | 2 | 3 | 3 | 3 | 1 | 2 | 1 | 3 | 3 | 1 | **G** |
| Weston et al. [20] | 1 | 1 | 1 | 1 | 2 | 3 | 3 | 3 | 1 | 1 | 1 | 3 | 2 | 1 | **G** |
| Caballero et al. [79] | 1 | 1 | 1 | 1 | 2 | 3 | 3 | 3 | 1 | 2 | 1 | 3 | 3 | 2 | **F** |
| Caballero et al. [17] | 1 | 1 | 1 | 1 | 2 | 3 | 3 | 3 | 1 | 2 | 1 | 3 | 3 | 2 | **F** |
| Castagna et al. [46] | 1 | 1 | 1 | 1 | 2 | 3 | 3 | 3 | 1 | 2 | 1 | 3 | 3 | 1 | **G** |
| Silva et al. [69] | 1 | 1 | 1 | 1 | 2 | 3 | 3 | 3 | 1 | 2 | 1 | 3 | 3 | 1 | **G** |
| Silva et al. [61] | 1 | 1 | 1 | 2 | 2 | 3 | 3 | 3 | 2 | 2 | 1 | 3 | 3 | 1 | **F** |
| DiSalvo et al. [115] | 1 | 2 | 3 | 2 | 2 | 3 | 3 | 3 | 1 | 3 | 1 | 3 | 3 | 1 | **P** |
| Weston et al. [100] | 1 | 1 | 1 | 1 | 2 | 3 | 3 | 3 | 1 | 2 | 1 | 3 | 3 | 1 | **G** |
| Weston et al. [106] | 1 | 1 | 1 | 2 | 2 | 3 | 3 | 3 | 1 | 2 | 1 | 3 | 3 | 1 | **F** |
| Weston et al. [116] | 1 | 1 | 1 | 1 | 2 | 3 | 3 | 3 | 1 | 2 | 1 | 3 | 3 | 1 | **G** |
| Barbero-Alvarez et al. [97] | 1 | 1 | 1 | 1 | 2 | 3 | 3 | 3 | 1 | 2 | 1 | 3 | 3 | 1 | **G** |
| Bizzini et al. [90] | 1 | 1 | 1 | 1 | 2 | 3 | 3 | 3 | 1 | 2 | 1 | 3 | 3 | 1 | **G** |
| Boullosa et al. [50] | 1 | 1 | 1 | 1 | 2 | 3 | 3 | 3 | 1 | 2 | 1 | 3 | 3 | 1 | **G** |
| Castagna et al. [8] | 1 | 1 | 1 | 2 | 2 | 3 | 3 | 3 | 1 | 2 | 1 | 3 | 3 | 1 | **F** |

| Study | Quality Assessment Tool for Observational Cohort and Cross-Sectional Studies checklist question number | | | | | | | | | | | | | | |
| --- | --- | --- | --- | --- | --- | --- | --- | --- | --- | --- | --- | --- | --- | --- | --- |
|  | 1 | 2 | 3 | 4 | 5 | 6 | 7 | 8 | 9 | 10 | 11 | 12 | 13 | 14 | Quality rating |
| Mallo et al. [117] | 1 | 1 | 1 | 1 | 2 | 3 | 3 | 3 | 1 | 2 | 1 | 3 | 3 | 1 | **G** |
| Stulp et al. [59] | 2 | 1 | 1 | 2 | 2 | 3 | 3 | 3 | 1 | 2 | 1 | 3 | 3 | 1 | **F** |
| Costa et al. [118] | 1 | 1 | 1 | 1 | 2 | 3 | 3 | 3 | 1 | 2 | 1 | 3 | 3 | 1 | **G** |
| Barbero-Alvarez et al. [119] | 1 | 1 | 1 | 1 | 2 | 3 | 3 | 3 | 2 | 2 | 1 | 3 | 3 | 1 | **F** |
| Casajus et al. [62] | 1 | 1 | 1 | 1 | 2 | 3 | 3 | 3 | 1 | 2 | 1 | 3 | 3 | 1 | **G** |
| Silva et al. [81] | 1 | 1 | 1 | 1 | 2 | 3 | 3 | 3 | 1 | 2 | 1 | 3 | 3 | 2 | **F** |
| Palmer et al. [86] | 1 | 1 | 1 | 1 | 2 | 3 | 3 | 3 | 1 | 2 | 1 | 3 | 3 | 1 | **G** |
| Pietraszewski et al. [91] | 1 | 1 | 1 | 1 | 2 | 3 | 3 | 3 | 1 | 2 | 1 | 3 | 3 | 2 | **F** |
| Teixeira et al. [72] | 1 | 1 | 1 | 1 | 2 | 3 | 3 | 3 | 1 | 2 | 1 | 3 | 3 | 1 | **G** |
| Martínez Reñon et al. [73] | 1 | 1 | 1 | 1 | 2 | 3 | 3 | 3 | 2 | 2 | 1 | 3 | 3 | 1 | **F** |
| Metz et al. [74] | 1 | 2 | 1 | 3 | 2 | 3 | 3 | 3 | 2 | 2 | 1 | 3 | 3 | 1 | **P** |
| Casajus et al. [63] | 1 | 1 | 1 | 2 | 1 | 3 | 3 | 3 | 1 | 1 | 1 | 3 | 2 | 1 | **G** |
| Castillo et al. [38] | 1 | 1 | 1 | 1 | 2 | 3 | 3 | 3 | 1 | 2 | 1 | 3 | 3 | 1 | **G** |
| Castillo et al. [81] | 1 | 1 | 1 | 1 | 2 | 3 | 3 | 3 | 1 | 2 | 1 | 3 | 3 | 1 | **G** |
| de Oliveira et al. [92] | 1 | 1 | 1 | 1 | 2 | 3 | 3 | 3 | 1 | 2 | 1 | 3 | 3 | 1 | **G** |
| Gomez-Carmona et al. [120] | 2 | 2 | 1 | 2 | 2 | 3 | 3 | 3 | 3 | 2 | 3 | 3 | 3 | 1 | **P** |
| Mazaheri et al. [82] | 1 | 1 | 1 | 1 | 2 | 3 | 3 | 3 | 2 | 2 | 2 | 3 | 3 | 1 | **P** |
| Paes et al. [70] | 1 | 1 | 1 | 1 | 2 | 3 | 3 | 3 | 1 | 2 | 1 | 3 | 3 | 1 | **G** |
| Yanci et al. [126] | 1 | 1 | 1 | 1 | 2 | 3 | 3 | 3 | 1 | 2 | 1 | 3 | 3 | 1 | **G** |
| Bozdogan et al. [77] | 1 | 1 | 1 | 2 | 2 | 3 | 3 | 3 | 2 | 2 | 1 | 3 | 3 | 1 | **P** |
| Castagna et al. [128] | 1 | 1 | 1 | 1 | 2 | 3 | 3 | 3 | 1 | 1 | 1 | 3 | 3 | 1 | **G** |
| Castillo et al. [121] | 1 | 1 | 1 | 1 | 2 | 3 | 3 | 3 | 1 | 2 | 1 | 3 | 3 | 1 | **G** |
| Dolanski et al. [122] | 1 | 1 | 1 | 1 | 2 | 3 | 3 | 3 | 1 | 2 | 2 | 3 | 3 | 1 | **F** |

| Study | Quality Assessment Tool for Observational Cohort and Cross-Sectional Studies checklist question number | | | | | | | | | | | | | | |
| --- | --- | --- | --- | --- | --- | --- | --- | --- | --- | --- | --- | --- | --- | --- | --- |
|  | 1 | 2 | 3 | 4 | 5 | 6 | 7 | 8 | 9 | 10 | 11 | 12 | 13 | 14 | Quality rating |
| Fernandez-Elias et al. [123] | 1 | 1 | 1 | 2 | 2 | 3 | 3 | 3 | 1 | 2 | 1 | 3 | 3 | 1 | **F** |
| Castagna et al. [83] | 1 | 1 | 1 | 1 | 2 | 3 | 3 | 3 | 1 | 2 | 1 | 3 | 3 | 1 | **G** |
| Castillo et al. [102] | 1 | 1 | 1 | 1 | 2 | 3 | 3 | 3 | 1 | 2 | 1 | 3 | 3 | 1 | **G** |
| Gianturco et al. [120] | 2 | 1 | 1 | 1 | 2 | 3 | 3 | 3 | 1 | 2 | 1 | 3 | 3 | 1 | **F** |
| Riiser et al. [70] | 1 | 1 | 1 | 1 | 2 | 3 | 3 | 3 | 1 | 2 | 1 | 3 | 3 | 1 | **G** |
| Sanchez-Garcia et al. [126] | 1 | 1 | 1 | 1 | 2 | 3 | 3 | 3 | 1 | 2 | 1 | 3 | 3 | 1 | **G** |
| Talovic et al. [77] | 1 | 1 | 1 | 1 | 2 | 3 | 3 | 3 | 1 | 2 | 2 | 3 | 3 | 1 | **F** |
| Banda et al. [60] | 1 | 1 | 1 | 2 | 2 | 3 | 3 | 3 | 1 | 2 | 2 | 3 | 3 | 1 | **P** |
| Casajus et al. [64] | 1 | 1 | 1 | 1 | 2 | 3 | 3 | 3 | 1 | 2 | 1 | 3 | 3 | 1 | **G** |
| Castagna et al. [84] | 1 | 1 | 1 | 1 | 2 | 3 | 3 | 3 | 1 | 2 | 1 | 3 | 3 | 1 | **G** |
| Castillo et al. [104] | 1 | 1 | 1 | 1 | 2 | 3 | 3 | 3 | 1 | 2 | 1 | 3 | 3 | 1 | **G** |
| Joo et al. [123] | 1 | 1 | 1 | 1 | 2 | 3 | 3 | 3 | 1 | 2 | 1 | 3 | 3 | 1 | **G** |
| Malaguti et al. [71] | 1 | 2 | 1 | 2 | 2 | 3 | 3 | 3 | 2 | 2 | 2 | 3 | 3 | 1 | **P** |
| Maslennikov et al. [55] | 2 | 2 | 1 | 2 | 2 | 3 | 3 | 3 | 2 | 2 | 2 | 3 | 3 | 1 | **P** |
| Santos-Silva et al. [85] | 2 | 1 | 1 | 1 | 2 | 3 | 3 | 3 | 1 | 2 | 1 | 3 | 1 | 1 | **F** |
| Gacek et al. [75] | 1 | 1 | 1 | 1 | 2 | 3 | 3 | 3 | 1 | 2 | 1 | 3 | 3 | 2 | **F** |
| Mascherini et al. [76] | 1 | 1 | 1 | 1 | 2 | 3 | 3 | 3 | 1 | 2 | 1 | 3 | 3 | 1 | **G** |
| McCarrick et al. [24] | 1 | 2 | 1 | 1 | 2 | 3 | 3 | 3 | 1 | 2 | 1 | 3 | 3 | 1 | **F** |

| Study | Quality Assessment Tool for Observational Cohort and Cross-Sectional Studies checklist question number | | | | | | | | | | | | | | |
| --- | --- | --- | --- | --- | --- | --- | --- | --- | --- | --- | --- | --- | --- | --- | --- |
|  | 1 | 2 | 3 | 4 | 5 | 6 | 7 | 8 | 9 | 10 | 11 | 12 | 13 | 14 | Quality rating |
| Munirogulu et al. [56] | 1 | 1 | 1 | 2 | 2 | 3 | 3 | 3 | 2 | 2 | 2 | 3 | 3 | 1 | **P** |
| Muscella et al. [16] | 1 | 1 | 1 | 1 | 2 | 3 | 3 | 3 | 1 | 2 | 1 | 3 | 1 | 1 | **G** |
| Petri et al. [66] | 1 | 1 | 1 | 1 | 2 | 3 | 3 | 3 | 2 | 2 | 1 | 3 | 1 | 1 | **F** |
| Aguilar et al. [95] | 1 | 1 | 1 | 1 | 2 | 3 | 3 | 3 | 2 | 2 | 1 | 3 | 1 | 1 | **G** |
| Castagna et al. [49] | 1 | 1 | 1 | 1 | 2 | 3 | 3 | 3 | 2 | 1 | 1 | 1 | 1 | 1 | **G** |
| Lopez-Garcia et al. [67] | 1 | 1 | 1 | 2 | 2 | 3 | 3 | 3 | 1 | 2 | 2 | 3 | 3 | 1 | **F** |
| Ozaeta et al. [124] | 1 | 1 | 1 | 1 | 1 | 3 | 3 | 3 | 1 | 2 | 1 | 3 | 3 | 1 | **G** |
| Romano et al. [127] | 1 | 1 | 1 | 1 | 2 | 3 | 3 | 3 | 1 | 2 | 1 | 3 | 3 | 1 | **G** |
| Martínez-Torremocha et al. [104] | 1 | 1 | 1 | 1 | 2 | 3 | 3 | 3 | 1 | 2 | 1 | 3 | 3 | 1 | **G** |
| Preissler et al. [41] | 1 | 1 | 1 | 1 | 1 | 3 | 3 | 3 | 1 | 2 | 1 | 3 | 3 | 1 | **G** |

1-yes 2 - no; 3 - cannot determine, not applicable or not reported; G - good; F - fair; P – poor. *Bizzini M, Junge A, Bahr R, Dvorak J. Female soccer referees selected for the FIFA Women's World Cup 2007: survey of injuries and musculoskeletal problems. Br J Sports Med. 2009;43(12):936-942.

| Criteria no. | Question |
| --- | --- |
| 1 | Was the study question or objective clearly stated? |
| 2 | Were eligibility/selection criteria for the study population prespecified and clearly described? |
| 3 | Were the participants in the study representative of those who would be eligible for the test/service/intervention in the general or clinical population of interest? |
| 4 | Were all eligible participants that met the prespecified entry criteria enrolled? |
| 5 | Was the sample size sufficiently large to provide confidence in the findings? |
| 6 | Was the test/service/intervention clearly described and delivered consistently across the study population? |
| 7 | Were the outcome measures prespecified, clearly defined, valid, reliable, and assessed consistently across all study participants? |
| 8 | Were the people assessing the outcomes blinded to the participants' exposures/interventions? |
| 9 | Was the loss to follow-up after baseline 20% or less? Were those lost to follow-up accounted for in the analysis? |
| 10 | Did the statistical methods examine changes in outcome measures from before to after the intervention? Were statistical tests done that provided p values for the pre-to-post changes? |
| 11 | Were outcome measures of interest taken multiple times before the intervention and multiple times after the intervention (i.e., did they use an interrupted time-series design)? |
| 12 | If the intervention was conducted at a group level (e.g., a whole hospital, a community, etc.) did the statistical analysis take into account the use of individual-level data to determine effects at the group level? |
|  | **Quality Rating (Good, Fair, or Poor)** |

**Supplementary Table 3.** Questions from the Quality Assessment Tool for Before-After (Pre-Post) Studies With No Control Group checklist used to evaluate methodological quality of the included articles.

**Legend:**

1-YES 2 - NO; 3 - cannot determine, not applicable or not reported

**Supplementary Table 3.** Results of methodological quality assessment for Before-After (Pre-Post) Studies With No Control Group.

| Study | Quality Assessment Tool for Before-After (Pre-Post) Studies With No Control Group checklist question number | | | | | | | | | | | | |
| --- | --- | --- | --- | --- | --- | --- | --- | --- | --- | --- | --- | --- | --- |
|  | 1 | 2 | 3 | 4 | 5 | 6 | 7 | 8 | 9 | 10 | 11 | 12 | Quality rating |
| Castillo et al. [79] | 1 | 3 | 2 | 1 | 2 | 1 | 1 | 3 | 1 | 1 | 2 | 2 | **F** |
| Castillo et al. [18] | 1 | 3 | 2 | 1 | 2 | 1 | 1 | 3 | 1 | 1 | 2 | 2 | **F** |
| Castillo et al. [94] | 1 | 3 | 2 | 1 | 2 | 1 | 1 | 3 | 1 | 1 | 2 | 2 | **F** |
| Coffi et al. [96] | 1 | 1 | 2 | 1 | 2 | 2 | 1 | 3 | 1 | 1 | 2 | 2 | **F** |
| Castillo et al. [105] | 1 | 3 | 2 | 1 | 2 | 2 | 1 | 3 | 1 | 1 | 2 | 2 | **F** |
| Schmidt et al. [110] | 1 | 1 | 2 | 2 | 1 | 1 | 1 | 3 | 1 | 1 | 1 | 2 | **F** |
| Meckel et al. [113] | 1 | 2 | 2 | 2 | 2 | 2 | 1 | 3 | 1 | 1 | 2 | 2 | **P** |
| Senecal et al. [116] | 1 | 1 | 2 | 1 | 2 | 1 | 1 | 3 | 1 | 1 | 2 | 2 | **F** |
| Baydemir et al. [118] | 1 | 2 | 2 | 1 | 2 | 2 | 1 | 3 | 1 | 1 | 2 | 2 | **P** |
| Fernandez-Ruiz et al. [119] | 1 | 1 | 2 | 1 | 2 | 1 | 1 | 3 | 1 | 1 | 2 | 2 | **F** |
|  |  |  |  |  |  |  |  |  |  |  |  |  |  |

1-yes 2 - no; 3 - cannot determine, not applicable or not reported; G - good; F - fair; P - poor

| Criteria no. | Question |
| --- | --- |
| 1 | Was the study described as randomized, a randomized trial, a randomized clinical trial, or an RCT? |
| 2 | Was the method of randomization adequate (i.e., use of randomly generated assignment)? |
| 3 | Was the treatment allocation concealed (so that assignments could not be predicted)? |
| 4 | Were study participants and providers blinded to treatment group assignment? |
| 5 | Were the people assessing the outcomes blinded to the participants' group assignments? |
| 6 | Were the groups similar at baseline on important characteristics that could affect outcomes (e.g., demographics, risk factors, co-morbid conditions)? |
| 7 | Was the overall drop-out rate from the study at endpoint 20% or lower of the number allocated to treatment? |
| 8 | Was the differential drop-out rate (between treatment groups) at endpoint 15 percentage points or lower? |
| 9 | Was there high adherence to the intervention protocols for each treatment group? |
| 10 | Were other interventions avoided or similar in the groups (e.g., similar background treatments)? |
| 11 | Were outcomes assessed using valid and reliable measures, implemented consistently across all study participants? |
| 12 | Did the authors report that the sample size was sufficiently large to be able to detect a difference in the main outcome between groups with at least 80% power? |
| 13 | Were outcomes reported or subgroups analyzed prespecified (i.e., identified before analyses were conducted)? |
| 14 | Were all randomized participants analyzed in the group to which they were originally assigned, i.e., did they use an intention-to-treat analysis? |
|  | **Quality Rating (Good, Fair, or Poor)** |
|  |  |

**Supplementary Table 3.** Questions from the Quality Assessment of Controlled Intervention Studies checklist used to evaluate methodological quality of the included articles.

**Legend:**

1-YES 2 - NO; 3 - cannot determine, not applicable or not reported

| Study | Quality Assessment of Controlled Intervention Studies checklist question number | | | | | | | | | | | | | | |
| --- | --- | --- | --- | --- | --- | --- | --- | --- | --- | --- | --- | --- | --- | --- | --- |
|  | 1 | 2 | 3 | 4 | 5 | 6 | 7 | 8 | 9 | 10 | 11 | 12 | 13 | 14 | Quality rating |
| Yanaoka et al. [100] | 1 | 3 | 3 | 3 | 3 | 3 | 1 | 1 | 1 | 1 | 1 | 1 | 1 | 1 | **F** |
| Muscella et al. [16] | 3 | 3 | 3 | 3 | 3 | 2 | 1 | 1 | 3 | 2 | 1 | 2 | 1 | 3 | **P** |
|  |  |  |  |  |  |  |  |  |  |  |  |  |  |  |  |

**Supplementary Table 3.** Results of methodological quality assessment for Controlled Intervention Studies.

1- yes 2 - no; 3 - cannot determine, not applicable or not reported; G - good; F - fair; P - poor
