# Supplementary material for: A Systematic Review of the Physical, Physiological, Nutritional and Anthropometric Profiles of Soccer Referees
Source: Sports Med Open. 2023 Aug 10;9:72. doi: 10.1186/s40798-023-00610-7 (PMC10415246; doi:10.1186/s40798-023-00610-7)
Supplement: Supplementary file 4 — Additional file 4: Table S4. Tests often used among soccer referees. [file 40798_2023_610_MOESM4_ESM.docx]

| Reference | Sample | Physical test |
| --- | --- | --- |
| Castagna and D’Ottavio [33] | 8 R | VO_2max_ using K2 technology (K2, Cosmed, Rome, Italy): progressive protocol running on an athletic track. |
| Krustrup and Bangsbo [34] | 22 R | YOYO intermittent recovery; VO_2max_ MedGraphics CPX/D breath-by-breath gas analysis system (Saint Paul, Minneapolis,  MN, USA): laboratory treadmill. |
| Castagna et al. [35] | 8 R | Progressive test running on an athletic track (velocity determined by an audio signal). |
| Castagna et al. [12] | 22 R | 50-m sprint; 200-m sprint; 12-minute run test. |
| Krustrup et al. [13] | 15 AR | VO_2max_ MedGraphics CPX/D breath-by-breath gas analysis system (Saint Paul, Minneapolis, MN, USA): laboratory treadmill; 12-minute run test; 2 × 50 m sprints. |
| Tessitore et al. [14] | 10 R | Countermovement jump and broad jump. |
| Mallo et al. [36] | 22 AR | 12-minute run test; 2 × 50 m sprints. |
| Mallo et al. [22] | 11 R | 6 × 40 m sprint test, with 1 minute 30 seconds recovery between each sprint; 150 m in 30 s and walking 50 m in 40 seconds, around the 400-m athletic track. |
| Weston et al. [37] | 17 R | 6 × 40 m sprint test, with 1 minute 30 seconds recovery between each sprint; 150 m in 30 s and walking 50 m in 35 seconds, around the 400-m athletic track. |
| Castagna et al. [8] | 245 AR | YOYO intermittent endurance; assistant referee intermittent endurance test, VO_2max_ MedGraphics CPX/D breath-by-breath gas analysis system (Saint Paul, Minneapolis, MN, USA: laboratory treadmill; 3 × 30 m with 90 seconds of recovery. |
| Boullosa et al. [50] | 16 R | Countermovement jump and YOYO intermittent recovery |
| Castillo et al. [38] | 12 R  8 AR | 3 × 30 m with 90 seconds of recovery with 90 seconds of recovery. |
| Castillo et al. [51] | 24 R | Countermovement jump; 2 × 30 m with 90 seconds of recovery. |
| Castillo et al. [52] | 18 FR  36 AR | Countermovement jump. |
| Risser et al. [39] | 9 FR  21 AR | Change of direction test; 5 × 30 m sprint test, with 30 seconds of recovery between each sprint; 6 × 40 m sprint test, with 60 seconds of recovery between each sprint. |
| Castillo et al. [40] | 25 FR  19 AR | Straight Line Sprint Test (3 × 30 m with 90 seconds of recovery); change of direction test; YOYO intermittent recovery. |
| Preissler et al. [41] | 14 R | 6 × 40 m sprint test with 1 minute of recovery period; 40 runs over  75 m with 15 seconds of recovery, interspersed with 25 m walking in 22 seconds. |

**Supplementary Table S4.** Tests often used among soccer referees.

R (referees); AR (assistant referees)
